# The Toxoplasma gondii Cyst Wall Interactome

**DOI:** 10.1128/mBio.02699-19

**Published:** 2020-02-04

**Authors:** Vincent Tu, Tadakimi Tomita, Tatsuki Sugi, Joshua Mayoral, Bing Han, Rama R. Yakubu, Tere Williams, Aline Horta, Yanfen Ma, Louis M. Weiss

**Affiliations:** a Department of Pathology, Albert Einstein College of Medicine, Bronx, New York, USA; b Department of Medicine, Albert Einstein College of Medicine, Bronx, New York, USA; University of Arizona

**Keywords:** BirA, cyst wall, *Toxoplasma gondii*, protein interactions, proteomics

## Abstract

A characteristic of the latent cyst stage of Toxoplasma gondii is a thick cyst wall that forms underneath the membrane of the bradyzoite vacuole. Previously, our laboratory group published a proteomic analysis of purified *in vitro* cyst wall fragments that identified an inventory of cyst wall components. To further refine our understanding of the composition of the cyst wall, several cyst wall proteins were tagged with a promiscuous biotin ligase (BirA*), and their interacting partners were screened by streptavidin affinity purification. Within the cyst wall pulldowns, previously described cyst wall proteins, dense granule proteins, and uncharacterized hypothetical proteins were identified. Several of the newly identified hypothetical proteins were validated to be novel components of the cyst wall and tagged with BirA* to expand the model of the cyst wall interactome. Community detection of the cyst wall interactome model revealed three distinct clusters: a dense granule, a cyst matrix, and a cyst wall cluster. Characterization of several of the identified cyst wall proteins using genetic strategies revealed that MCP3 affects *in vivo* cyst sizes. This study provides a model of the potential protein interactions within the cyst wall and the groundwork to understand cyst wall formation.

## Abstract (precis)

A model of the cyst wall interactome was constructed using proteins identified through BioID. The proteins within this cyst wall interactome model encompass several proteins identified in a prior characterization of the cyst wall proteome. This model provides a more comprehensive understanding of the composition of the cyst wall and may lead to insights on how the cyst wall is formed.

## INTRODUCTION

Toxoplasma gondii, an opportunistic pathogen, infects more than 1 billion people worldwide. Most infected individuals harbor this intracellular parasite as a latent infection with cysts (containing bradyzoites) being found in the central nervous system. For the most part, those with a healthy immune system do not present with symptoms of toxoplasmosis; however, these cysts can be reactive in immunocompromised hosts, leading to active retinochoroiditis or encephalitis (1). Currently, there are no drugs that can eliminate T. gondii cysts from infected patients (2).

Latent infection with T. gondii is characterized by the unique presence of a cyst wall that lies underneath the membrane of the cyst vacuole. The cyst wall is an electron-dense structure that consists of an outer condensed sponge-like layer that transitions into a looser layer extending into the cyst matrix (3). Proteins such as MAG1 (4), MCP4 (5), BCP1 (6), and various dense granule proteins (7) have been shown to localize to the cyst wall. In addition, various proteins and sugars have been characterized to play important roles in cyst wall biology (8–13). For example, deletion of CST1, BPK1, or GRA6 resulted in a reduction of cysts recovered from murine brains. While some proteins within the cyst wall have been characterized, the molecular mechanism of cystogenesis remains unknown.

Recently, an initial characterization of the cyst wall proteome of Pru Δ*ku80* Δ*hxgprt* (Pru) was completed using tandem mass spectrometry of immunoprecipitated *in vitro* cyst wall membrane fragments (14). This analysis yielded an inventory of previously undescribed cyst wall proteins (CST2/GRA50, CST3/GRA51, CST4, CST5/GRA52, and CST6/GRA53) that were validated to localize to the cyst wall by immunofluorescence. Two of these novel cyst wall proteins were further characterized, which revealed that CST2 plays a role in parasite virulence. The identification of additional cyst wall proteins has provided further insight into the composition of the cyst wall; however, whether and how these proteins are important for the formation of the cyst wall remain unknown. In addition, the molecular interactions of the components at the cyst wall remain unclear.

To refine the model of the cyst wall structure, the interacting partners of various cyst wall proteins were investigated using BioID, a method that uses a promiscuous biotin ligase for proximity-based biotinylation (15). This led to the identification of additional hypothetical proteins that were further validated to localize to the cyst wall. Analysis of an interactome model based on the proteins identified by this BioID study revealed distinct protein communities which may reflect unique protein interaction clusters within the cyst wall. Lastly, uncharacterized cyst wall proteins were investigated for their roles in parasite replication, virulence, cyst formation, cyst dimensions, and cyst morphology.

## RESULTS

### Identification of interacting protein candidates of known cyst wall proteins.

**FIG 1:**
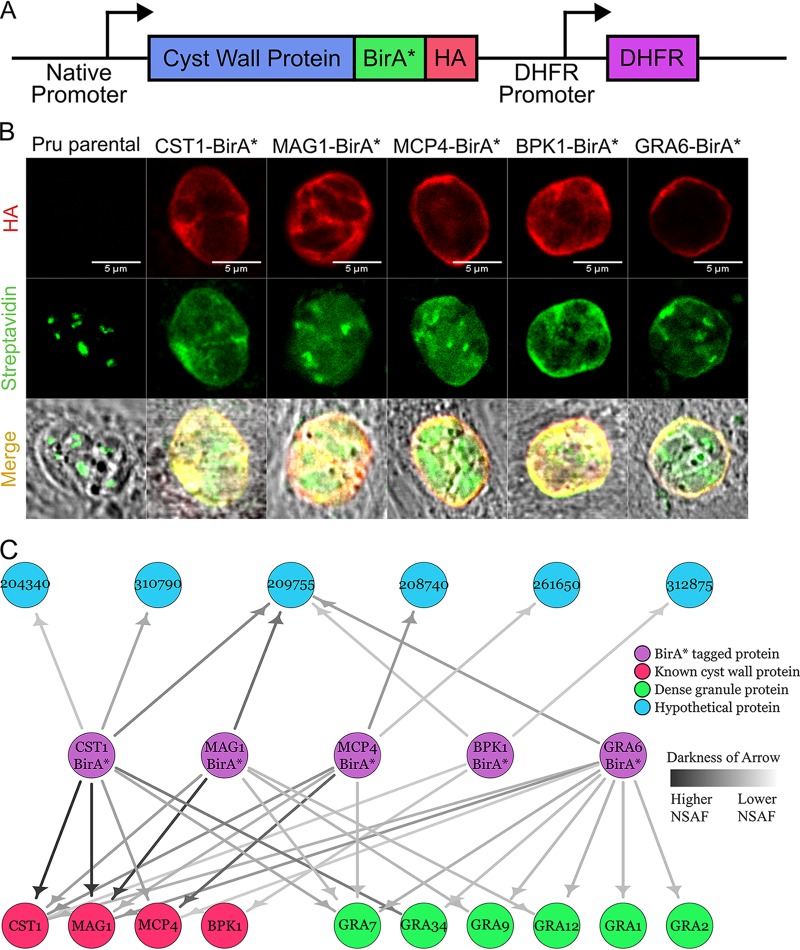
Initial cyst wall pulldowns with BioID reveal hypothetical proteins. (A) Schematic diagram of the exogenous expression construct containing the promoter and coding sequence of the cyst wall gene of interest fused C terminally with BirA* and 3×HA followed by the DHFR selectable marker. (B) Immunofluorescence micrographs of Pru and BirA*-tagged parasites (CST1, MAG1, MCP4, BPK1, and GRA6) stained with anti-HA antibody and Alexa Fluor 488-conjugated streptavidin under pH 8 conditions supplemented with 150 μM biotin. Endogenously biotinylated proteins within the apicoplast are observed within these parasite strains. (C) Network graph showing potential parasite-specific interacting proteins of each BirA*-tagged cyst wall protein (CST1, MAG1, MCP4, BPK1, and GRA6) identified during the bradyzoite stage. BirA* cyst wall bait proteins, known cyst wall proteins, dense granule proteins, and hypothetical proteins are represented by purple, red, green, and blue nodes, respectively. Darker arrows correspond to higher normalized spectral abundance factor (NSAF) values of prey proteins identified in each pulldown.

The previously identified cyst wall proteins CST1 (11), BPK1 (12), MCP4 (5), MAG1 (4), and GRA6 (13) were tagged on their C termini with a promiscuous biotin ligase (BirA*) (15) followed by a 3-tandem hemagglutinin tag (3×HA) and independently expressed as a second copy transgene under their native promoters in Pru parasites (Fig. 1A). Localization of these transgenes under bradyzoite culture conditions was assessed by immunofluorescence assays (IFAs) to ensure that the BirA* tag did not cause improper localization. Colocalization of the HA signal from each transgene was observed at the cyst wall with CST1 serving as the cyst wall marker (see Fig. S1A in the supplemental material), demonstrating that the BirA* tag did not disrupt cyst wall localization of these proteins. Next, the activity of BirA* at the cyst wall was assessed by IFA and immunoblot. Under bradyzoite-inducing conditions without supplemental biotin, the BirA* tag fused to BPK1, MCP4, MAG1, or GRA6 showed no activity (no additional biotin signal above the endogenously biotinylated proteins of the apicoplast was observed under IFA or immunoblot) (Fig. S1B and C); however, the CST1-BirA* strain showed self-biotinylation of the CST1 protein (the CST1-BirA* bradyzoite lane without supplemental biotin revealed a biotinylated band corresponding to the high molecular weight of CST1) (Fig. S1C). This band likely corresponds to the increased biotinylated signal observed at the cyst wall without exogenous biotin (Fig. S1B, CST1-BirA* panel). When exogenous 150 μM biotin was supplemented to the bradyzoite-inducing medium, the BirA* tag fused to MAG1, MCP4, BPK1, CST1, and GRA6 displayed biotinylation activity as an increase in streptavidin signal that was observed at the cyst wall compared to that in the Pru strain under IFA (Fig. 1B). Increased signals from streptavidin were also observed on the immunoblot in lanes containing BirA*-tagged parasites differentiated in biotin-supplemented medium (Fig. S1C).

10.1128/mBio.02699-19.1 FIG S1 BirA*-tagged proteins correctly localize to the cyst wall. (A) Immunofluorescence images showing that BirA*-tagged parasites (CST1, MAG1, MCP4, BPK1, and GRA6) localize correctly to the cyst wall as seen with colocalization with CST1. (B) Immunofluorescence assay of Pru and BirA*-tagged parasites stained with anti-HA antibody and Alexa Fluor 488-conjugated streptavidin under pH 8 conditions without exogenous biotin added to the medium. Endogenously biotinylated proteins within the apicoplast are observed within these parasite strains. (C) Western blot of lysates corresponding to Pru and BirA*-tagged parasites differentiated in pH 8 supplemented with and without 150 μM biotin was probed with streptavidin-horseradish peroxidase (HRP). ALD1 was used as the loading control. Download FIG S1, TIF file, 2.8 MB. Copyright © 2020 Tu et al. 2020 Tu et al. This content is distributed under the terms of the Creative Commons Attribution 4.0 International license.

After confirming the activity of BirA* at the cyst wall, the targets labeled by each BirA* transgene were affinity captured with streptavidin beads and identified by liquid chromatography-tandem mass spectrometry (LC-MS/MS). After accounting for host cell proteins, for well-characterized and predicted nonsecreted proteins, and for endogenously biotinylated proteins from the Pru sample, a network graph was constructed showing the potential interacting proteins of each BirA*-tagged cyst wall protein (Fig. 1C). Each BirA* strain revealed a list of unique and common protein interaction candidates that consisted of known cyst wall proteins, dense granule proteins, and hypothetical proteins. Dense granule proteins known to associate with the cyst wall such as GRA1 (7), GRA2 (9), GRA7 (7), GRA9 (9, 12), GRA12, and GRA34 (14) were identified within these pulldowns. Some of the hypothetical proteins identified were recently validated cyst wall proteins such as CST4 (TgME49_261650) and MCP3 (TgME49_208740) (14). Hypothetical protein TgME49_209755 corresponds to a bradyzoite matrix protein called MAG2 which was identified in a separate study (V. Tu, J. Mayoral, R. Yakubu, T. Tomita, T. Sugi, B. Han, T. Williams, Y. Ma, and L. M. Weiss, submitted for publication). While hypothetical protein TgME49_204340 was also discovered in the cyst wall proteome (14), it was not assessed whether it localized to the cyst wall. In the tachyzoite stage, hypothetical protein TgME49_204340 has been shown to localize to the apical/subapical region of the parasite (note its previous gene identifier [ID] was 20.m03858) (16). Hypothetical protein TgME49_310790 has been demonstrated to localize to the dense granule and the tachyzoite parasitophorous lumen (note its previous gene ID was TgME49_110790) (17). Whether these proteins localized to the cyst wall under bradyzoite conditions remained unknown. Lastly, the localization of hypothetical protein TgME49_312875 has not been investigated. These initial pulldowns identified potential interacting partners of the BirA*-tagged cyst wall proteins.

### Expansion of the cyst wall interactome model through novel cyst wall proteins.

**FIG 2:**
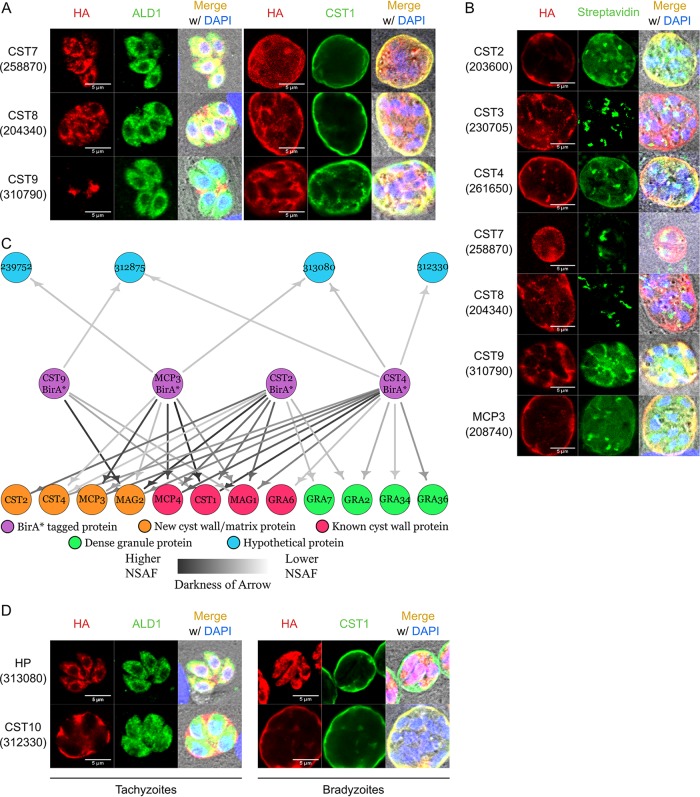
Iterative BioID pulldowns of cyst wall proteins identify a novel cyst wall protein. (A) Immunofluorescence images of tagged hypothetical proteins stained with anti-HA and anti-ALD1 (cytosolic marker) or anti-CST1 (cyst wall marker) antibodies. Tachyzoites: HA in red and ALD1 in green; bradyzoites: HA in red and CST1 in green. (B) Immunofluorescence micrographs of endogenously BirA*-tagged parasites (CST2, CST3, CST4, CST7, CST8, CST9, and MCP3) stained with anti-HA antibody and Alexa Fluor 488-conjugated streptavidin under pH 8 conditions supplemented with 150 μM biotin. (C) Network graph showing potential parasite-specific interacting proteins of each BirA*-tagged cyst wall protein (CST2, CST4, CST9, and MCP3). BirA* cyst wall bait proteins, known cyst wall proteins, novel cyst wall proteins, dense granule proteins, and hypothetical proteins are represented by purple, red, orange, green, and blue nodes, respectively. Darker arrows correspond to higher normalized spectral abundance factor (NSAF) values of prey proteins identified in each pulldown. (D) Immunofluorescence images of HA-tagged hypothetical proteins (HP) identified from the group 2 BirA* pulldowns stained with anti-HA and anti-ALD1 (cytosolic marker) or anti-CST1 (cyst wall marker) antibodies. Tachyzoites: HA in red and ALD1 in green; bradyzoites: HA in red and CST1 in green.

To assess whether hypothetical proteins TgME49_204340, TgME49_310790, TgME49_312875, and TgME49_258870 (a top hit candidate from the cyst wall proteome [14]) localized to the cyst wall under bradyzoite conditions, these genes were tagged at their endogenous loci on their C termini with BirA*-3×HA using CRISPR/Cas9, using previously described methods (14). These proteins were tagged with BirA*, as it does not disrupt cyst wall localization (Fig. S1A) and allows for a subsequent round of BirA* pulldowns if these proteins localized to the cyst wall. The resulting transgenic parasites were stained by IFA and, under pH 7 conditions, hypothetical proteins TgME49_258870, TgME49_204340, and TgME49_310790 localized within the tachyzoite body or were secreted to the parasitophorous vacuole (Fig. 2A, left). Under bradyzoite induction conditions, the BirA*-tagged TgME49_258870, TgME49_204340, and TgME49_310790 proteins localized to the cyst wall as demonstrated by their colocalization with CST1 (Fig. 2A, right). Due to their cyst wall localizations, TgME49_258870, TgME49_204340, and TgME49_310790 were renamed CST7, CST8, and CST9, respectively. Attempts to tag hypothetical protein TgME49_312875 were unsuccessful. To further expand the cyst wall interactome model, recently validated cyst wall proteins CST2, CST3, CST4, and MCP3 (14) were also tagged endogenously at their C termini with BirA*-3×HA.

Of these group 2 BioID tagged constructs (CST2, CST3, CST4, CST7, CST8, CST9, and MCP3), only the CST2-BirA*, CST4-BirA*, CST9-BirA*, and MCP3-BirA* parasites showed additional biotinylation signals at the cyst wall when these parasites were differentiated with exogenous biotin supplemented in the high alkaline medium (Fig. 2B). The BirA* tags on CST3, CST7, and CST8 did not exhibit biotinylation activity, possibly due to protein folding making the tag inaccessible. When no exogenous biotin was provided, CST2-BirA*, CST4-BirA*, CST9-BirA*, and MCP3-BirA* strains showed no increase in streptavidin signal at the cyst wall (see Fig. S2A).

10.1128/mBio.02699-19.2 FIG S2 Intracellular and extracellular immunofluorescence of BirA*- and HA-tagged cyst wall proteins. (A) Immunofluorescence assay of BirA*-tagged parasites differentiated to bradyzoites without exogenous biotin added to the induction medium. Fixed cells containing cyst vacuoles were stained with anti-HA antibody and Alexa Fluor 488-conjugated streptavidin, which labels the endogenously biotinylated proteins within the apicoplast. (B) Immunofluorescence micrographs of extracellular BirA*- (CST9) or HA-tagged (CST10) tachyzoites stained with anti-HA (red), anti-GRA1 (green), and DAPI (4′,6-diamidino-2-phenylindole; blue). Download FIG S2, TIF file, 2.8 MB. Copyright © 2020 Tu et al. 2020 Tu et al. This content is distributed under the terms of the Creative Commons Attribution 4.0 International license.

Since only CST2-BirA*, CST4-BirA*, CST9-BirA*, and MCP3-BirA* parasites showed biotin ligase activity at the cyst wall, streptavidin affinity capture of the potential interacting partners of these cyst wall strains was performed and processed by LC-MS/MS. The proteins identified from this subsequent round of BirA* pulldowns contained well-characterized cyst wall proteins, recently validated cyst wall proteins, dense granule proteins, and additional hypothetical proteins (Fig. 2C). GRA36 was discovered previously in the cyst wall proteome (14). The novel hypothetical proteins identified from the group 2 BirA* pulldowns included TgME49_239752, TgME49_312330, TgME49_312875, and TgME49_313080. Since TgME49_239752 contains an endoplasmic reticulum retention signal at its C terminus and its function is predicted to be a glycosyltransferase, its localization and function were not further pursued in this study. Hypothetical protein TgME49_312875, which also appeared in the group 1 BirA* pulldowns (Fig. 1C), reappeared in group 2 data sets.

To assess whether hypothetical proteins (HP) TgME49_313080 and TgME49_312330 localized to the cyst wall, they were tagged endogenously on their C termini with a 1×HA tag. IFA analysis showed that TgME49_313080 localized within tachyzoites around the nucleus and also extended into the basal end of the parasite that seems to connect the parasites together, reminiscent of the intravacuolar connection between tachyzoites (18). After bradyzoite differentiation, TgME49_313080 remained within the bradyzoite body (Fig. 2D, top). Hypothetical protein TgME49_312330 localized to the lumen of the tachyzoite parasitophorous vacuole and to the cyst wall under bradyzoite conditions (Fig. 2D, bottom). Interestingly, TgME49_312330 was also identified within the cyst wall proteome; however, its abundance within the Pru strain compared to that in the ΔCST1 strain was just under the threshold (14). Due to its cyst wall localization, TgME49_312330 was renamed CST10. To investigate whether CST10 was secreted from the dense granules, extracellular parasites were fixed and stained with anti-GRA1 and anti-HA antibodies. Using CST9 (TgME49_310790) as a positive control for dense granule staining (17), anti-HA signal within CST10-1×HA parasites was not observed (Fig. S2B). This iterative expansion of the cyst wall interactome model revealed proteins reciprocally identified from both BirA* pulldowns and identified another novel cyst wall protein.

### Analysis of the model of the cyst wall interactome.

**FIG 3:**
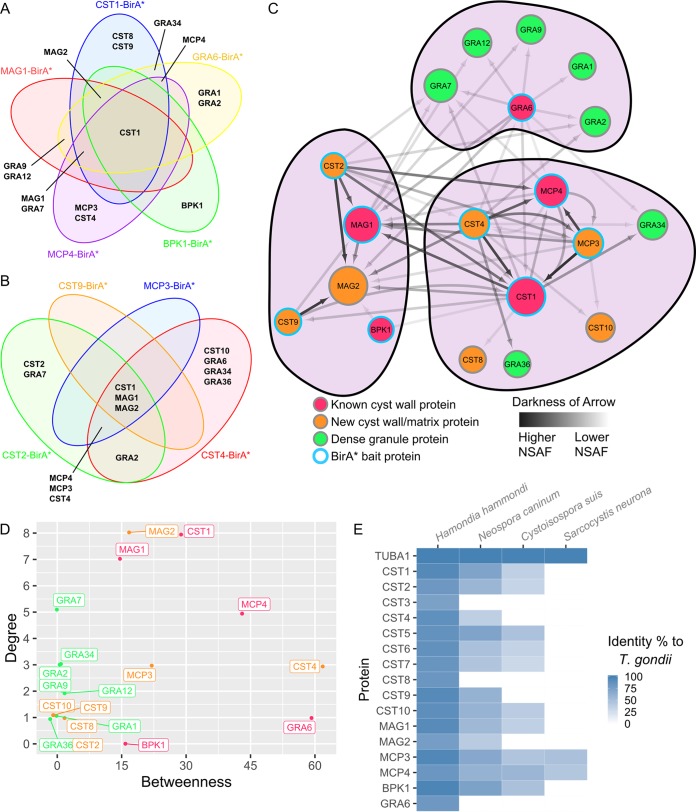
Cyst wall interactome model analysis reveals distinct clusters. Venn diagrams showing the proteins that are common between the group 1 (A) or group 2 (B) cyst wall BirA* pulldowns. Each BirA* pulldown is represented by a different colored circle. (C) Network of the proteins identified in the group 1 and group 2 BirA* pulldowns. BirA* bait protein nodes are represented by a blue outline. Known cyst wall proteins, dense granule proteins, and novel cyst wall/matrix proteins are represented by red, green, and orange nodes, respectively. Darkness of arrows represents the normalized spectral abundance factor (NSAF) of the prey proteins identified in each pulldown. Size of each node represents the in-degrees or the amount of times a protein was identified within each pulldown. Protein clustering was performed using the Louvain method of community detection. (D) Graph of each node’s betweenness, which signifies the extent to which nodes stand between each other, versus their in-degrees, the frequency of each node appearing in a pulldown. (E) Heat map showing the percentage of identity between alpha tubulin and each T. gondii cyst wall and cyst matrix protein to their orthologs in other cyst-forming coccidians.

In the group 1 BirA* pulldowns with CST1-BirA*, BPK1-BirA*, MAG1-BirA*, MCP4-BirA*, and GRA6-BirA* parasites, CST1 was the most prominent protein identified among all five BirA* cyst wall proteins (Fig. 3A). Following CST1, MAG2 was found in the pulldowns of all these BirA*-tagged cyst wall proteins aside from MCP4-BirA*. This may be due to MAG2 and MCP4’s mutually exclusive localization to the cyst matrix and cyst wall, respectively. Next, GRA7 and MAG1 may interact with CST1-BirA*, MAG1-BirA*, MCP4-BirA*, and GRA6-BirA* but not BPK1-BirA*. While a lack of interaction between GRA7 and BPK1 was previously observed (12), Buchholz et al. did observe an interaction between BPK1 and proteins MAG1 and MCP4 using coimmunoprecipitation. However, these interactions may result from these proteins binding to larger complexes which were not captured by BPK1-BirA*. Interestingly, GRA6-BirA* captured all dense granule proteins identified within the group 1 BirA* pulldowns, while the other BirA* proteins showed various potential interactions with dense granule proteins. Finally, CST8 and CST9, CST4 and MCP3, and BPK1 were found only in the CST1-BirA*, MCP4-BirA*, and BPK1-BirA* pulldowns, respectively.

For the group 2 BirA* pulldowns, CST1, MAG1, and MAG2 were identified in the CST2-BirA*, CST4-BirA*, CST9-BirA*, and MCP3-BirA* data sets (Fig. 3B). On the other hand, MCP3, MCP4, and CST4 were identified in pulldowns with CST2-BirA*, CST4-BirA*, and MCP3-BirA* but not with CST9-BirA*. While GRA2 was identified in both the CST2-BirA* and CST4-BirA* pulldowns, CST2, GRA7, CST10, GRA6, GRA34, and GRA36 were identified within only single data sets in the group 2 BirA* pulldowns.

By analyzing the proteins identified between each pulldown, some reciprocal protein pairs between baits were identified. These include CST1-MCP4, CST1-MAG1, CST1-CST9, MCP4-CST4, MCP4-MCP3, and MCP3-CST4 pairings. Interestingly, while CST2-BirA* identified CST1, MAG1, MCP3, MCP4, and CST4 within its pulldown, peptides from CST2 were found only within its own data set. The absence of CST2 within other BirA* pulldowns may be due to a detection limit of CST2 if this protein is not very abundant within the cyst wall or if CST2 only interacts with the N termini of these other bait proteins, which may be too far from the C-terminally located BirA* tag.

To incorporate all these pulldowns together, a network graph was constructed using the normalized spectral abundance factor (NSAF) values obtained from each BirA* bait’s prey (Fig. 3C). Nodes CST1, MAG2, MAG1, and MCP4 have the largest size within this interactome network model, signifying that they were the most common proteins identified within all BirA* pulldowns. In addition, these four nodes generally had darker arrows pointed to them, signifying their abundance within this interactome model, even after normalizing for protein molecular weight. Using Louvain analysis (an algorithm for detecting communities in networks), clustering of the network revealed three distinct clusters. In general, the dense granule proteins, cyst matrix proteins (around MAG1 and MAG2), and cyst wall proteins (around CST1, CST4, MCP3, and MCP4) formed their own communities. These clusters were generated using a weighted undirect graph with loops (self-identifying peptides) removed.

Analysis of the cyst wall interactome model revealed that CST1, MAG1, and MAG2 have the highest in-degrees between all proteins, signifying that they were the most common proteins identified from all cyst wall BirA* pulldowns (Fig. 3D). Betweenness analysis, which shows the importance of a node in connecting the different clusters together, suggests the data from the CST4-BirA* and GRA6-BirA* pulldowns are most important in structuring the three distinct clusters seen within the cyst wall interactome model. Amino acid sequence analysis using blastp and multiple-sequence alignments revealed that the protein sequences of T. gondii cyst wall and matrix proteins are most homologous to their Hammondia hammondi counterparts (Fig. 3E; Fig. S3 provides a phylogenetic tree for these data). The analyses of this network provide a more comprehensive overview on the composition of the T. gondii cyst wall and the potential protein interactions within this structure.

10.1128/mBio.02699-19.3 FIG S3 Multiple-sequence alignment tree diagram of cyst wall and cyst matrix proteins. Protein sequences of Toxoplasma gondii cyst wall and cyst matrix proteins and alpha tubulin were aligned to their orthologous proteins within Hammondia hammondi, Neospora caninum, Cystoisospora suis, and Sarcocystis neurona, based on protein identity. The similarity between each protein was visualized using a phylogenetic tree. Download FIG S3, TIF file, 0.9 MB. Copyright © 2020 Tu et al. 2020 Tu et al. This content is distributed under the terms of the Creative Commons Attribution 4.0 International license.

### Characterization of novel cyst wall proteins.

**FIG 4:**
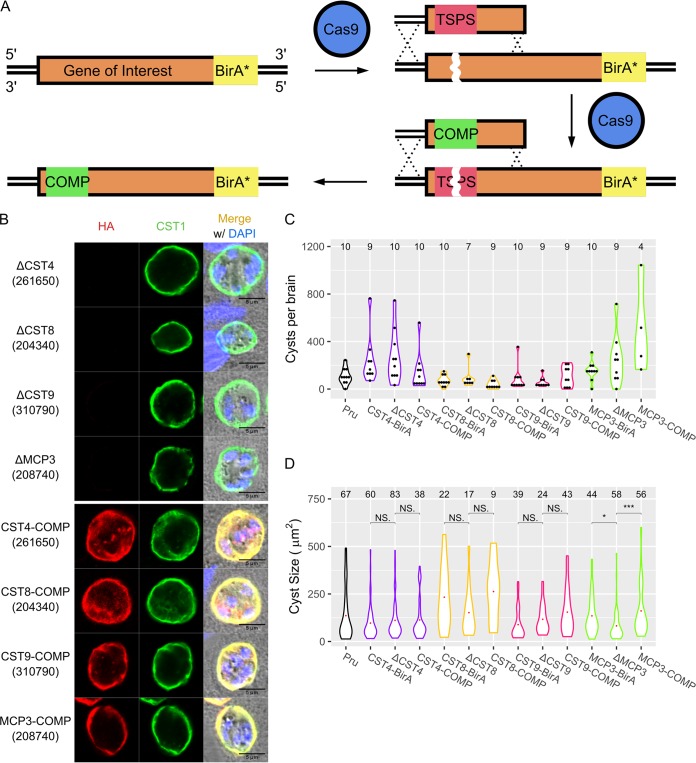
Characterization of cyst wall proteins reveals MCP3 affects cyst size. (A) Schematic showing the endogenous editing of genomic loci using a targetable stop PAM sequence (TSPS) to introduce a Cas9-targetable sequence of premature stop codons. Complementation of the edited loci was performed by targeting the premature stop codons with a TSPS-specific sgRNA and replacing the TSPS with synonymous mutations of the coding region. The red region indicates the TSPS sequence, while the green region indicates synonymous mutations. (B) IFA showing CST4, CST8, CST9, and MCP3 knockout (top) and complement (bottom) bradyzoites stained with anti-HA (red) and anti-CST1 (green) antibodies. (C) Brain cyst counts of mice infected with Pru, CST4, CST8, CST9, and MCP3 knockout and complement parasite strains at 30 days postinfection. Numbers of brains counted are listed above each violin plot. No significant differences in cyst numbers between BirA*, knockout, and complement parasites in each strain group were observed (*P* > 0.05). (D) Size analysis of *in vivo* cysts obtained from Pru, CST4, CST8, CST9, and MCP3 knockout and complement parasite strains. Numbers of cysts imaged and analyzed are listed above each violin plot. NS, not significant; *, *P* < 0.05; ***, *P* < 0.001.

To characterize the role of cyst wall proteins in cyst biology, single guide RNAs (sgRNAs) targeting the N termini of CST4, CST8, CST9, and MPC3 BirA* parasites were cotransfected with donor DNA oligonucleotides into their respective endogenously BirA*-tagged parental strains using a deletion strategy described previously (14). Premature stop codons at the N termini of CST8 and CST9 were successfully introduced using this strategy (confirmed by sequence data, as shown in Fig. S4). Surprisingly, CST4 and MCP3 deletion parasites still expressed signal from anti-HA antibodies under IFA even after the insertion of premature stop codons into their N termini was validated by sequencing (data not shown). The lack of a gene knockout was likely due to an incorrect gene model for these two genes, with their actual start codons occurring downstream of the predicted start codon. Therefore, a different sgRNA targeting more downstream of their start codon was designed. When searching for protospacer adjacent motif (PAM) sequences to knockout these genes that were further downstream, no other PAM sequences adjacent to the knockout PAM sequence were identified. This posed an issue, as there would be only one PAM sequence to generate the deletion strains but not to complement the gene back. To circumvent this problem, donor DNA containing a stretch of stop codons followed by a PAM sequence was constructed so that this sequence can be uniquely targeted by its own sgRNA (see Table S2). This targetable stop PAM sequence (TSPS) allowed for ΔCST4 and ΔMCP3 parasites to be created, followed by their complemented strains (Fig. 4A; TSPS was confirmed by sequence data as shown in Fig. S4). IFA showed that cyst wall proteins CST4, CST8, CST9, and MCP3 were successfully knocked out and complemented back (Fig. 4B).

10.1128/mBio.02699-19.4 FIG S4 Sanger sequencing results of the loci targeted by Cas9 of ΔCST4, ΔCST8, ΔCST9, and ΔMCP3 parasites. Chromatograms showing the sequencing results of the N termini of ΔCST8 and ΔCST9 parasites demonstrating the inserted premature stop codons. ΔCST4 and ΔMCP3 parasites had the targetable stop PAM sequence (TSPS) inserted further downstream from the start codon. The red boxes depict the DNA sequences where stop codons or TSPSs were introduced to generate the knockout strains. Download FIG S4, JPG file, 2.0 MB. Copyright © 2020 Tu et al. 2020 Tu et al. This content is distributed under the terms of the Creative Commons Attribution 4.0 International license.

10.1128/mBio.02699-19.8 TABLE S2 Oligonucleotides used as donor DNA to generate HA-tagged, knockout, and complement strains. Mutations are designated in red, with stop codons designated as lowercase letters and the HA sequence in green. Download Table S2, DOCX file, 0.1 MB. Copyright © 2020 Tu et al. 2020 Tu et al. This content is distributed under the terms of the Creative Commons Attribution 4.0 International license.

To investigate whether CST4, CST8, CST9, and MCP3 play roles in cyst biology, the respective BirA*, knockout and complement strains were injected into C57BL/6 mice. No significant differences in cyst numbers between the deletion strain and the parental or complement strains for CST4, CST8, CST9, and MCP3 in murine brains were observed (Fig. 4C). However, when the area of the cysts recovered from mouse brains was analyzed, ΔMCP3 parasites generated smaller cysts than the parental and complement strains, suggesting MCP3 plays a role in *in vivo* cyst growth (Fig. 4D). Tachyzoite replication between ΔCST4, ΔCST8, ΔCST9, and ΔMCP3 parasites and their parental and complement counterparts was not significantly different (see Fig. S5A). Next, the localization of cyst wall proteins CST1, MAG1, and MCP4 as well as the α-*N*-acetylgalactosamine sugar pattern on the cyst wall (as detected by fluorescein-conjugated Dolichos biflorus agglutinin lectin) of these cyst wall protein knockouts was assessed by IFA. Within ΔCST4, ΔCST8, ΔCST9, and ΔMCP3 *in vitro* cysts, no mislocalization or abnormal staining of CST1, MAG1 (Fig. S5B), MCP4, or α-*N*-acetylgalactosamine (Fig. S5C) was observed. Lastly, the survival of mice infected with the cyst wall knockout strains was also investigated. No significant difference was observed between the survival rates of mice infected with the Pru strain and those with the knockout strains (Fig. S5D). While cyst wall proteins CST4, CST8, CST9, and MCP3 were not found to have a phenotype in these studies of cystogenesis, MCP3 had a clearly defined phenotype that affected cyst growth and size (Fig. 4D).

10.1128/mBio.02699-19.5 FIG S5 Additional characterization of cyst wall proteins. (A) Quantification of the plaque size from each parasite strain. The number of plaques analyzed per strain is provided above each violin plot. No significant differences in plaque size between the parental, knockout, and complement strains were observed for each cyst wall protein. Immunofluorescence images of cyst wall protein knockout parasites stained for CST1 and MAG1 (B) or CST, DBA, and MCP4 (C) under bradyzoite conditions *in vitro*. (D) Survival curve of C57BL/6 mice challenged with 100,000 Pru, ΔCST4, ΔCST8, ΔCST9, and ΔMCP3 parasites (*n* = 10). Download FIG S5, TIF file, 1.8 MB. Copyright © 2020 Tu et al. 2020 Tu et al. This content is distributed under the terms of the Creative Commons Attribution 4.0 International license.

### Ultrastructural analysis on the morphology of cyst wall protein knockout parasites.

**FIG 5:**
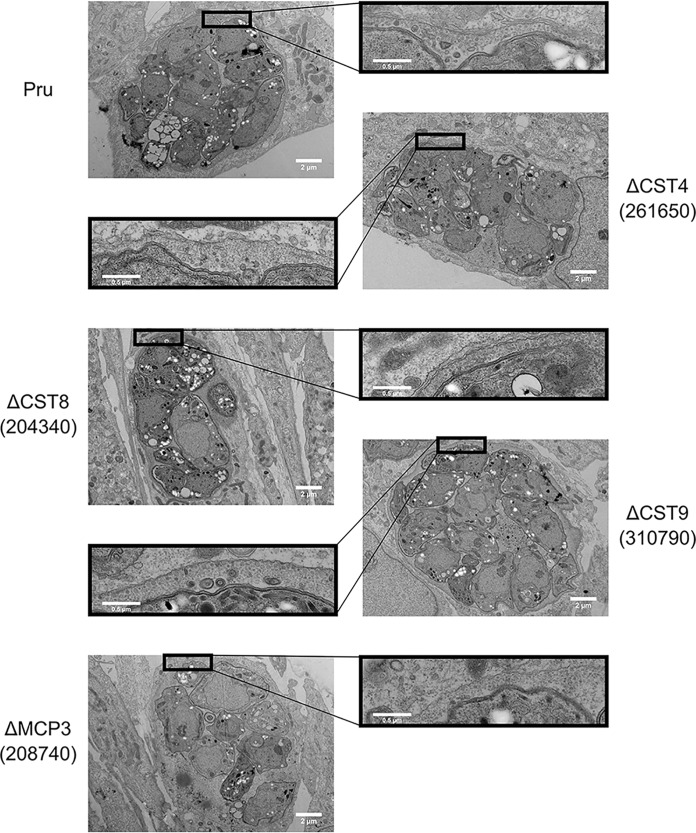
Novel cyst wall proteins do not affect morphology of cyst ultrastructure. Transmission electron micrographs of *in vitro* cysts from the Pru, ΔCST4, ΔCST8, ΔCST9, and ΔMCP3 parasite strains.

To investigate whether CST4, CST8, CST9, and MCP3 affect the cyst wall ultrastructure, *in vitro* cysts of the respective knockout parasites were prepared for transmission electron microscopy (EM). No distinguishing differences in cyst morphology were observed within *in vitro* cysts of ΔCST4, ΔCST8, ΔCST9, and ΔMCP3 parasites compared to those of the Pru grand parental strain (Fig. 5). To observe the ultrastructural localization of these cyst wall proteins, *in vitro* cysts of BirA*- or HA-tagged CST4, CST8, CST9, CST10, and MCP3 parasites were prepared for immuno-EM analysis. Staining with anti-HA antibody showed that the localization of these novel cyst wall proteins occurred at the cyst wall (see Fig. S6). While these cyst wall proteins were localized to the cyst wall, they did not affect the morphology of the cyst when deleted.

10.1128/mBio.02699-19.6 FIG S6 Ultrastructural localization of cyst wall proteins. Immunoelectron micrographs of *in vitro* cysts from Pru and BirA*-tagged parasites (CST4, CST8, CST10, and MCP3) showing labeling with the anti-HA antibody. White arrows point to particles from the gold-conjugated anti-rat antibody. Download FIG S6, TIF file, 2.6 MB. Copyright © 2020 Tu et al. 2020 Tu et al. This content is distributed under the terms of the Creative Commons Attribution 4.0 International license.

## DISCUSSION

To further refine the model of the cyst wall proteome, a network of potential cyst wall-interacting components was constructed using BioID through various cyst wall proteins. By tagging well-characterized cyst wall proteins with BirA*, a network of cyst wall protein pulldowns was generated (Fig. 1C) which was subsequently expanded by a second round of BirA* pulldowns (Fig. 2C). The final interactome network (Fig. 3C) provides a model of how proteins within the cyst wall structure may be interacting (although it should be appreciated that a limitation of BioID is that this interaction is defined by proximity labeling rather than via direct binding to a partner protein). Within the interactome model, it can be appreciated that CST1, MAG1, and MCP4 are the central components of each cyst wall pulldown. While MAG2 was a protein that appeared frequently within the cyst wall pulldowns, its interaction with cyst wall proteins may be mainly nonspecific, as it displays an exclusive cyst matrix localization and does not play a role in cystogenesis or in cyst growth (Tu et al., submitted). In contrast, the characterization on cyst biology of parasites lacking CST1 (11) or MAG1 (B. Han, T. Tomita, R. Yakubu, V. Tu, J. Mayoral, T. Sugi, Y. Ma, and L. M. Weiss, submitted for publication) showed that these two proteins are important for cystogenesis, reinforcing the model that these two proteins may be crucial components of the cyst wall interactome. Additionally, analysis of the cyst wall interactome model revealed three distinct protein clusters, which may reflect unique protein interaction clusters within the cyst wall. However, these interactions will need to be further validated through independent experiments that examine directly protein-protein interactions (e.g., yeast two-hybrid and other studies), as it is possible that the proteins identified from this network are proximal proteins rather than interacting proteins. By generating a network of the potential cyst wall interactions using BioID, a better understanding of how the components of the cyst wall may be interacting with one another can be gained.

BioID has been a useful tool to characterize and identify components of various T. gondii structures (15, 19–21). The promiscuous biotin ligase used in this study has been described to function within an ∼10-nm range (22); therefore, proteins that interact with the BirA*-tagged baits outside this range might not be detected. For example, proteins CST3, CST5, CST6, CST7, GRA3, and GRA14 were detected in the cyst wall proteome (14) but not within the interactome. This may be due to these proteins being part of the membrane that could be pulled down with the anti-CST1 antibody but not within 10 nm of any of the proteins that had a BirA* label. Nine cyst wall proteins were used for this interactome study, since not all the proteins tagged with BirA* displayed functional activity (Fig. 2B). Utilization of other cyst wall proteins in BioID would likely expand the interactome model, providing additional proteins that could be included in the cyst wall protein inventory. These reasons probably underlie why only ∼38% of the top candidates within the cyst wall proteome were identified within the cyst wall interactome model using the BioID strategy. In addition to T. gondii-specific proteins, human proteins were also identified within all of the pulldowns (see Data Set S1 in the supplemental material). However, upon analysis of the proteins from the host cell, most of the human proteins consisted of endogenously biotinylated proteins, histone proteins, heat shock proteins, and cytoskeletal or ribosomal proteins. These human proteins may largely be nonspecific proteins that associated with the pulldown material.

10.1128/mBio.02699-19.9 DATA SET S1 LC-MS/MS results obtained from the group 1 and group 2 BirA* pulldowns. Numbers indicate the NSAF values obtained from Scaffold4 software after all the pulldown data were merged together. Nonspecific proteins from the Pru strain were filtered out from each pulldown. Proteins highlighted in red are the hypothetical proteins identified in this study. Group 1 pulldowns were duplicated and pooled before analysis. Group 2 pulldowns were performed once. Download Data Set S1, XLS file, 0.5 MB. Copyright © 2020 Tu et al. 2020 Tu et al. This content is distributed under the terms of the Creative Commons Attribution 4.0 International license.

The new cyst wall proteins characterized within this study include CST4 (TgME49_261650), CST8 (TgME49_204340), CST9 (TgME49_310790), and MCP3 (TgME49_208740). Of these proteins, CST8, CST9, and MCP3 also appear within tachyzoite GRAomes (19, 21). CST8 and CST9 are predicted to contain signal peptides, while CST8 and CST10 (TgME49_312330) have predicted transmembrane domains. CST7 (TgME49_258870), which was not further characterized in this study, also contains predicted transmembrane domains. Hypothetical protein TgME49_312875, which appeared in both rounds of BirA* pulldowns, shares homology to serine/arginine repetitive matrix protein 2 and microtubule-associated protein Futsch. Similar to what has been seen with CST1 and MAG1, we found that CST7, -8, -9, and -10 also display some degree of tachyzoite parasitophorous vacuole matrix localization but localize to the cyst wall and matrix in the bradyzoite stage. In addition, these cyst wall proteins have moderate to high levels of expression in both tachyzoite and bradyzoite stages based on their transcriptomic data on ToxoDB.

Studies on T. gondii AP2 transcription factors revealed that the expression of CST4 and MCP3, among other cyst wall and cyst matrix proteins such as BPK1, MCP4, and MAG2, was altered when the AP2 bradyzoite activators and repressors were disrupted. For example, parasites with deleted bradyzoite activators AP2XI-4 and AP2IV-3 showed a downregulation of CST4 (23) and MCP3 (24) compared to that of their parental strains following high alkaline stress. In the case of bradyzoite repressors, overexpression of AP2IX-9 (25) repressed expression of CST4 and MCP3, while deletion of AP2IV-4 (26) upregulated these two cyst wall genes. In addition, a gene regulatory network analysis assigned CST4 and MCP3 to the community of genes upregulated during the bradyzoite stage (27). Other genes within this community include BPK1, MAG1, CST10, MAG2, ENO1, LDH2, ANK1, and P-type ATPase. CST4 and MCP3 are among several cyst wall and matrix proteins that have their expression regulated during bradyzoite differentiation, directly or indirectly through AP2 transcription factors.

Initial attempts to target CST4 and MCP3 at their N termini and insert stop codons to stop their protein expressions were unsuccessful. Therefore, further downstream regions of CST4 and MCP4 were targeted using the TSPS which allows for accessible complementation at the site where stop codons were introduced. This method circumvents the previous issues of locating two adjacent PAM sites that could be targeted by efficient sgRNAs for deletion and complementation. Since downstream targeting of CST4 and MCP3 were successful in deleting these two genes, the actual start codons for these genes are likely downstream. The current gene model for CST4 (TgME49_261650) is likely two separate genes, as the ortholog equivalent of this gene in Hammondia hammondi is split into two coding sequences (HHA_261650 as the upstream coding sequence and HHA_461840 as the downstream coding sequence). Analysis of the mass spectrometry data on TgME49_261650 revealed no peptide sequences that mapped to the N-terminal regions of this protein. In addition, immunoblot analysis (data not shown) of CST4-BirA* parasites showed that the endogenously tagged CST4 has a lower molecular weight than its predicted gene model after accounting for the BirA* and 3×HA tags. These data support that TgME49_261650 is likely two genes and that the CST4-BirA* transgene corresponds to the downstream gene.

After generating the deletion and complementation strains for CST4, CST8, CST9, and MCP3, these parasites were assayed for parasite and cyst biology. Plaque assays show that these genes were not crucial for parasite replication (Fig. S5A), and survival assays show these genes were not important for parasite virulence (Fig. S5D). While these proteins localize to the cyst wall (Fig. S6), they are individually not critical for cystogenesis, as the number of cysts generated from the deletion strains did not differ from those by their parental or complement strains (Fig. 4C). This may be due to compensatory mechanisms among these various cyst wall proteins. Further investigations of the role of these cyst wall proteins on cystogenesis will need to include multiple gene knockouts to evaluate the effects of disruptions of multiple CST proteins on cyst formation.

While CST4, CST8, and CST9 do not seem to play a role in determining cyst size, a lack of MCP3 resulted in statistically smaller cysts (Fig. 4D). The reduction in size of ΔMCP3 cysts may be due to a slowing of bradyzoite replication; however, since a cyst’s packing density is inversely correlated with the volume of a cyst (28), it is possible that the decrease in ΔMCP3’s cyst size may not be directly linked to bradyzoite replication. Furthermore, tachyzoite replication between ΔMCP3 parasites and their parental and complement counterparts was not significantly different (Fig. S5A). It is possible that further studies on the dynamics of bradyzoite replication (28) in the ΔMCP3 strain may help elucidate the role of MCP in determining cyst size.

The cyst wall interactome model was constructed using the type II Pru strain with 3 days of pH 8 *in vitro* bradyzoite culture differentiation. While this time point was sufficient in achieving >90% bradyzoite induction, longer differentiation periods may reveal different compositions of proteins within the cyst wall. In addition, proteins within the cyst wall interactome model may vary depending on the parasite strain used. Future studies on this interactome model will require using the ME49 strain which can generate more cysts within mice, which will allow for the characterization of proteins within an *in vivo* cyst wall. In addition, crystal structure characterization of cyst wall proteins, super resolution microscopy of the localization of the various cyst wall proteins, and cryoEM analysis of the cyst wall complex will aid in understanding the formation of the cyst wall. With modern cryoEM techniques, macro structures of hundreds of mega Daltons have been solved (29). Combining the data from the cyst wall interactome model with these studies will further elucidate how the cyst wall is formed by T. gondii.

## MATERIALS AND METHODS

### Parasite culture.

Pru Δ*ku80* Δ*hxgprt* (Pru) wild-type parasites expressing superfolder green fluorescent protein (GFP) under the LDH2 promoter (14) and all subsequent parasite strains generated from this parental line in this study were cultured in human foreskin fibroblast (HFF) (ATCC:CRL-1634; Hs27) host cells, as described previously (14). For bradyzoite differentiation, parasites were inoculated into a confluent HFF monolayer and cultured for 2 h at 37°C in a 5% CO_2_ incubator. After 2 h, the medium was changed to pH 8.2 Dulbecco’s modified Eagle medium (DMEM) containing 50 mM HEPES without NaHCO_3_, supplemented with 1% fetal bovine serum (FBS), penicillin, and streptomycin. Following the medium change, parasites were incubated in a humid 37°C incubator without CO_2_ for 3 days with the bradyzoite differentiation medium being changed every other day.

### Immunofluorescence assay.

HFF monolayers were infected with epitope-tagged parasite tachyzoites and cultured for 24 h before fixing or were induced to become bradyzoites for 3 days before fixing. Fixed cells were stained by immunofluorescence as described previously (14). HA-tagged proteins were detected by an anti-HA rat monoclonal antibody 3F10 (Sigma; 1:500), parasite cytosol was detected by an anti-ALD1 rabbit antibody (1:500) (a kind gift from Kentaro Kato, University of Tokyo) (30), biotinylated proteins were detected by Alexa Fluor 488-conjugated streptavidin (Thermo Fisher; 1:500), and the cyst wall was detected by a salmonE anti-CST1 antibody (1:500) (11), anti-MCP4 mouse monoclonal antibody (1:500), Dolichos biflorus agglutinin (DBA)-fluorescein isothiocyanate (FITC) (1:500), or anti-MAG1 mouse monoclonal antibody (1:500). CF405M-conjugated anti-rabbit, Alexa Fluor 594-conjugated anti-rat or anti-mouse, or Alexa Fluor 488-conjugated anti-rabbit or anti-mouse antibodies were used as secondary antibodies (1:500). For dense granule staining, extracellular parasites were fixed with 4% paraformaldehyde (PFA) and permeabilized with 0.1% Triton X-100 before incubation with anti-HA rat monoclonal antibody 3F10 (1:250) and anti-GRA1 mouse monoclonal antibody 92.10B (1:500) (31). Mounted coverslips were imaged using a Leica TCS SP5 confocal microscope.

### Generation of BirA*-tagged cyst wall protein parasite strains.

The genomic locus containing the promoter region (1 kb to 2 kb upstream of the start codon) and the coding sequence (excluding the stop codon) of BPK1, MAG1, MCP4, and GRA6 was cloned upstream of the BirA* sequence (15) in frame into the pLIC-3HA-DHFR plasmid backbone (32). Forty micrograms of the resulting plasmid was cut with PsiI and transfected into Pru tachyzoites.

For genes encoding CST1, CST2, CST3, CST4, CST7, CST8, CST9, and MCP3, 0.4 kb to 1 kb of each gene’s C-terminal sequence was cloned in frame upstream of the BirA* sequence followed by the 3×HA epitope tag, HXGPRT 3′ untranslated region (UTR), dihydrofolate reductase (DHFR) selectable marker, and 0.4 kb to 1 kb of the gene’s 3′ UTR by HiFi assembly (NEB). Forty micrograms of the resulting construct was cut with SwaI to remove the pUC19 backbone before being transfected into Pru tachyzoites to endogenously tag the C termini of the genes through homologous recombination. Transfected parasites were selected with 1 μM pyrimethamine before being subcloned by limiting dilution as described elsewhere (14). A list of primers used is available in Table S1 in the supplemental material.

10.1128/mBio.02699-19.7 TABLE S1 Primers used in this study. Overhanging regions for Gibson assembly or KLD reaction are designated in green. Download Table S1, DOCX file, 0.1 MB. Copyright © 2020 Tu et al. 2020 Tu et al. This content is distributed under the terms of the Creative Commons Attribution 4.0 International license.

### Mass spectrometry identification of the interacting proteins of BirA*-tagged proteins.

Pulldown of the biotinylated proteins using high-capacity streptavidin agarose resins (Pierce) was performed as previously described (15). Briefly, Pru and BirA*-tagged parasite strains were inoculated into two 150-mm dishes with confluent HFF cells at a multiplicity of infection (MOI) of 1. After incubation with the differentiation medium supplemented with 150 μM biotin for 3 days, infected plates were washed three times with phosphate-buffered saline (PBS) containing a protease inhibitor cocktail (Roche). Then, the infected cells were scraped, pelleted, resuspended in RIPA buffer (50 mM Tris [pH 7.5], 150 mM NaCl, 0.1% SDS, 0.5% sodium deoxycholate, 1% NP-40, protease inhibitor cocktail), and sonicated at 20% amplitude for 30 cycles. The resulting lysate was centrifuged to remove insoluble material, and the supernatant was incubated with equilibrated streptavidin beads overnight at 4°C with rotation. The streptavidin beads were washed five times with RIPA buffer and three times with urea buffer (50 mM Tris-HCl [pH 7.5], 8 M urea, 150 mM NaCl) before being prepared for mass spectrometry analysis.

Biotinylated proteins on the streptavidin beads were reduced, alkylated, and digested by Lys-C and trypsin proteases. The peptide mixture was desalted using C_18_ tips (Thermo Fisher) and separated by liquid chromatography (nanoLC; Dionex NCS-3500RS Nano Rapid Separation system), and peptide masses and sequences were determined with the Thermo Scientific LTQ Orbitrap Velos mass spectrometer (nanoLC-MS/MS). Peptide masses were determined from the MS1 survey scan (*m/z* 300 to 1,600 at a resolution of 30 K), and the top 5 most abundant ions with charge states greater than 2 were selected for fragmentation using high-energy collisional dissociation (HCD). Peptides/proteins were identified using Mascot (version 2.5) against T. gondii (type II ME49 strain genome from ToxoDB version 12) and a human protein database (UniProt) with search parameters of peptide and product mass tolerances of 50 ppm and 0.4 Da, fixed modifications of carbamidomethyl cysteines, and variable modifications of deamidation (Asn and Gln), oxidation (Met), and phosphorylation (Ser, Thr, and Tyr). Scaffold (version 4) was used for peptide and protein validation.

### Cyst wall interactome model analysis.

Individual rounds of BirA* network graphs were generated in R using the igraph package. Weighted edges for the network were normalized using the NSAF values for each BirA* pulldown. Duplicate data for the group 1 BirA* pulldown were pooled. The combined interactome network was generated using the program Cytoscape. Communities of the network were analyzed by the Louvain method of community detection, which uses a greedy optimization method to optimize the modularity of the network. This analysis was performed on an undirected graph with self-loops removed. Betweenness and in-degrees of a node within the combined interactome network were plotted using ggplot2. Venn diagrams were plotted with the package VennDiagram in R. Protein identities between orthologous cyst wall proteins across cyst-forming coccidians were obtained using blastp. The identity percentage between different cyst-forming coccidians was graphed as a heat map within R using the package ggplot2. Protein alignment of cyst wall and cyst matrix proteins was also performed in R using the msa, ape, and ggtree packages.

### Endogenous deletion and complementation using a targetable stop PAM sequence.

For CST8 and CST9, these genes were knocked out and complemented using methods mentioned elsewhere (14). For CST4 and MCP3, p-HXGPRT-Cas9-GFP containing single guide RNAs (sgRNAs) targeting regions upstream of these genes’ C termini was prepared. Donor DNA oligonucleotides (Thermo Fisher) consisting of forward and reverse sequences of a 20-bp targetable stop PAM sequence (TSPS) and a sequence of tandem stop codons followed by a Cas9 protospacer adjacent motif (PAM) sequence flanked by the gene’s coding sequence were cotransfected with 20 μg of circular p-HXGPRT-Cas9-GFP at a 100:1 mol ratio into the appropriate BirA*-tagged parasite lines.

To complement the genes back into the knockout parasites, a sgRNA targeting the TSPS was cloned into p-HXGPRT-Cas9-GFP, and the resulting plasmid was cotransfected with donor DNA oligonucleotides containing synonymous mutations of the original coding sequence to reverse the premature stop codons. After transfection, transgenic parasites were selected with 25 μg/ml mycophenolic acid and 50 μg/ml xanthine for 10 days before subcloning immediately. Lists of primers and oligonucleotides used are available in Tables S1 and S2, respectively.

### Plaque assay.

A 27-gauge needle was used to lyse parasites from host cells, which were filtered through a 5-μm filter to remove host cell debris. Fifty parasites of the respective strains were added to three wells in six-well plates containing confluent HFFs. After 14 days, the infected monolayer was fixed and stained with a 20% methanol-0.5% crystal violet solution. Plaque sizes were imaged and determined using ImageJ.

### Survival assay cyst number and size.

Six-week-old female C57BL/6 mice (The Jackson Laboratory) were infected with 10^3^ or 10^5^ tachyzoites of the appropriate strains intraperitoneally (i.p.) and monitored every day. After 30 days, infected mice were sacrificed for brain cyst counts. Harvested brains were homogenized in PBS using a Wheaton Potter-Elvehjem tissue grinder with a 100- to 150-μm clearance (Thermo Fisher). The homogenate was observed under a Microphoto-FXA epifluorescence microscope (Nikon), and images of GFP fluorescent cysts were captured with a 60× lens objective for cyst size analysis using ImageJ. To calculate the size of a cyst, a microscope stage micrometer was imaged with a 60× lens objective, and the distance per pixel was determined with ImageJ, which was used to set the scale. Fluorescent cyst images were then converted to 32-bit images, and a threshold of the fluorescence from the whole cyst was applied. Then, the area of the threshold was measured to obtain the area of a cyst.

### Morphological and immunoelectron microscopy.

For ultrastructural analyses under transmission electron microscopy, *in vitro* cysts from Pru Δ*ku80* Δ*hxgprt*, ΔCST4, ΔCST8, ΔCST9, and ΔMCP3 parasites were prepared by incubating under bradyzoite-inducing conditions for 7 days. Cysts were fixed with 2.5% glutaraldehyde-2% paraformaldehyde in 0.1 M sodium cacodylate buffer, postfixed with 1% osmium tetroxide followed by 2% uranyl acetate, dehydrated through a graded series of ethanol, and embedded in LX112 resin (LADD Research Industries, Burlington, VT). Ultrathin sections were cut on a Leica Ultracut UC7, stained with uranyl acetate followed by lead citrate, and viewed on a JEOL 1400EX transmission electron microscope at 120 kV.

For immunoelectron microscopy analysis, *in vitro* Pru Δ*ku80* Δ*hxgprt*, CST4-BirA*, CST8-BirA*, CST10-BirA*, and MCP3-BirA* parasites were differentiated to bradyzoites for 7 days before fixing and were stained for immunofluorescence as described elsewhere (14). The HA-tagged cyst wall proteins were detected by incubating the cysts with an anti-HA rabbit monoclonal antibody C29F4 (Cell Signaling Technology; 1:500) followed by a goat anti-rabbit antibody conjugated with Alexa Fluor 488 and 10 nm colloidal gold (Thermo Fisher; 1:30). The stained samples were processed for electron microscopy analysis as described above.

### Statistical analysis.

Cyst numbers and cyst sizes were graphed in R as violin plots using ggplot2. To compare multiple groups, one-way analysis of variance (ANOVA) was performed followed by *post hoc* Tukey’s honestly significant difference (HSD) test. The R packages survminer and survival were used to compare the survival curves of mice infected with different parasite strains. The Gehan-Wilcox test was used to look for significance between the survival curves.

### Ethics statement.

All mouse experiments were conducted according to guidelines from the United States Public Health Service Policy on Humane Care and Use of Laboratory Animals. Animals were maintained in an AAALAC-approved facility, and all protocols were approved by the Institutional Care Committee of the Albert Einstein College of Medicine, Bronx, NY (animal welfare assurance no. A3312-01).

### Data availability.

The proteomic data from this paper have been deposited at ToxodB.org (EuPathdB.org).

## References

[1] Porter SB, Sande MA 1992 Toxoplasmosis of the central nervous system in the acquired immunodeficiency syndrome. N Engl J Med 327:1643–1648. doi:10.1056/NEJM199212033272306. 1359410

[2] Antczak M, Dzitko K, Długońska H 2016 Human toxoplasmosis-searching for novel chemotherapeutics. Biomed Pharmacother 82:677–684. doi:10.1016/j.biopha.2016.05.041. 27470411

[3] Lemgruber L, Lupetti P, Martins-Duarte ES, De Souza W, Vommaro RC 2011 The organization of the wall filaments and characterization of the matrix structures of *Toxoplasma gondii* cyst form. Cell Microbiol 13:1920–1932. doi:10.1111/j.1462-5822.2011.01681.x. 21899696

[4] Parmley SF, Yang S, Harth G, Sibley LD, Sucharczuk A, Remington JS 1994 Molecular characterization of a 65-kilodalton *Toxoplasma gondii* antigen expressed abundantly in the matrix of tissue cysts. Mol Biochem Parasitol 66:283–296. doi:10.1016/0166-6851(94)90155-4. 7808478

[5] Buchholz KR, Fritz HM, Chen X, Durbin-Johnson B, Rocke DM, Ferguson DJ, Conrad PA, Boothroyd JC 2011 Identification of tissue cyst wall components by transcriptome analysis of *in vivo* and *in vitro Toxoplasma gondii* bradyzoites. Eukaryot Cell 10:1637–1647. doi:10.1128/EC.05182-11. 22021236 PMC3232729

[6] Milligan-Myhre K, Wilson SK, Knoll LJ 2016 Developmental change in translation initiation alters the localization of a common microbial protein necessary for *Toxoplasma* chronic infection. Mol Microbiol 102:1086–1098. doi:10.1111/mmi.13538. 27671212 PMC5161674

[7] Ferguson D 2004 Use of molecular and ultrastructural markers to evaluate stage conversion of *Toxoplasma gondi*i in both the intermediate and definitive host. Int J Parasitol 34:347–360. doi:10.1016/j.ijpara.2003.11.024. 15003495

[8] Tu V, Yakubu R, Weiss LM 2018 Observations on bradyzoite biology. Microbes Infect 20:466–476. doi:10.1016/j.micinf.2017.12.003. 29287987 PMC6019562

[9] Caffaro CE, Koshy AA, Liu L, Zeiner GM, Hirschberg CB, Boothroyd JC 2013 A nucleotide sugar transporter involved in glycosylation of the *Toxoplasma* tissue cyst wall is required for efficient persistence of bradyzoites. PLoS Pathog 9:e1003331. doi:10.1371/journal.ppat.1003331. 23658519 PMC3642066

[10] Tomita T, Sugi T, Yakubu R, Tu V, Ma Y, Weiss LM 2017 Making home sweet and sturdy: *Toxoplasma gondii* ppGalNAc-Ts glycosylate in hierarchical order and confer cyst wall rigidity. mBio 8:e02048-16. doi:10.1128/mBio.02048-16. 28074022 PMC5225312

[11] Tomita T, Bzik DJ, Ma YF, Fox BA, Markillie LM, Taylor RC, Kim K, Weiss LM 2013 The *Toxoplasma gondi*i cyst wall protein CST1 is critical for cyst wall integrity and promotes bradyzoite persistence. PLoS Pathog 9:e1003823. doi:10.1371/journal.ppat.1003823. 24385904 PMC3873430

[12] Buchholz KR, Bowyer PW, Boothroyd JC 2013 Bradyzoite pseudokinase 1 is crucial for efficient oral infectivity of the *Toxoplasma gondii* tissue cyst. Eukaryot Cell 12:399–410. doi:10.1128/EC.00343-12. 23291621 PMC3629768

[13] Fox BA, Falla A, Rommereim LM, Tomita T, Gigley JP, Mercier C, Cesbron-Delauw M-F, Weiss LM, Bzik DJ 2011 Type II *Toxoplasma gondii* KU80 knockout strains enable functional analysis of genes required for cyst development and latent infection. Eukaryot Cell 10:1193–1206. doi:10.1128/EC.00297-10. 21531875 PMC3187049

[14] Tu V, Mayoral J, Sugi T, Tomita T, Han B, Ma YF, Weiss LM, Tu V, Mayoral J, Sugi T, Tomita T, Han B, Ma YF, Weiss LM 2019 Enrichment and proteomic characterization of the cyst wall from *in vitro Toxoplasma gondii* cysts. mBio 10:e00469-19. doi:10.1128/mBio.00469-19. 31040239 PMC6495374

[15] Chen AL, Kim EW, Toh JY, Vashisht AA, Rashoff AQ, Van C, Huang AS, Moon AS, Bell HN, Bentolila LA, Wohlschlegel JA, Bradley PJ 2015 Novel components of the *Toxoplasma* inner membrane complex revealed by BioID. mBio 6:e02357-14. doi:10.1128/mBio.02357-14. 25691595 PMC4337574

[16] Huynh M-H, Carruthers VB 2009 Tagging of endogenous genes in a *Toxoplasma gondii* strain lacking Ku80. Eukaryot Cell 8:530–539. doi:10.1128/EC.00358-08. 19218426 PMC2669203

[17] Lorestani A, Ivey FD, Thirugnanam S, Busby MA, Marth GT, Cheeseman IM, Gubbels M-J 2012 Targeted proteomic dissection of *Toxoplasma* cytoskeleton sub-compartments using MORN1. Cytoskeleton (Hoboken) 69:1069–1085. doi:10.1002/cm.21077. 23027733 PMC3566231

[18] Frénal K, Jacot D, Hammoudi P-M, Graindorge A, Maco B, Soldati-Favre D 2017 Myosin-dependent cell-cell communication controls synchronicity of division in acute and chronic stages of *Toxoplasma gondii*. Nat Commun 8:15710. doi:10.1038/ncomms15710. 28593938 PMC5477499

[19] Nadipuram SM, Kim EW, Vashisht AA, Lin AH, Bell HN, Coppens I, Wohlschlegel JA, Bradley PJ 2016 *In vivo* biotinylation of the *Toxoplasma* parasitophorous vacuole reveals novel dense granule proteins important for parasite growth and pathogenesis. mBio 7:e00808-16. doi:10.1128/mBio.00808-16. 27486190 PMC4981711

[20] Long S, Anthony B, Drewry LL, Sibley LD 2017 A conserved ankyrin repeat-containing protein regulates conoid stability, motility and cell invasion in *Toxoplasma gondii*. Nat Commun 8:2236. doi:10.1038/s41467-017-02341-2. 29269729 PMC5740107

[21] Pan M, Li M, Li L, Song Y, Hou L, Zhao J, Shen B 2019 Identification of novel dense-granule proteins in *Toxoplasma gondii* by two proximity-based biotinylation approaches. J Proteome Res 18:319–330. doi:10.1021/acs.jproteome.8b00626. 30362762

[22] Kim DI, Birendra KC, Zhu W, Motamedchaboki K, Doye V, Roux KJ 2014 Probing nuclear pore complex architecture with proximity-dependent biotinylation. Proc Natl Acad Sci U S A 111:E2453–E2461. doi:10.1073/pnas.1406459111. 24927568 PMC4066523

[23] Walker R, Gissot M, Croken MM, Huot L, Hot D, Kim K, Tomavo S 2013 The *Toxoplasma* nuclear factor TgAP2XI-4 controls bradyzoite gene expression and cyst formation. Mol Microbiol 87:641–655. doi:10.1111/mmi.12121. 23240624 PMC3556193

[24] Hong D-P, Radke JB, White MW 2017 Opposing transcriptional mechanisms regulate *Toxoplasma* development. mSphere 2:e00347-16. doi:10.1128/mSphere.00347-16. 28251183 PMC5322347

[25] Radke JB, Lucas O, De Silva EK, Ma Y, Sullivan WJ, Weiss LM, Llinas M, White MW 2013 ApiAP2 transcription factor restricts development of the *Toxoplasma* tissue cyst. Proc Natl Acad Sci U S A 110:6871–6876. doi:10.1073/pnas.1300059110. 23572590 PMC3637731

[26] Radke JB, Worth D, Hong D, Huang S, Sullivan WJ, Wilson EH, White MW 2018 Transcriptional repression by ApiAP2 factors is central to chronic toxoplasmosis. PLoS Pathog 14:e1007035. doi:10.1371/journal.ppat.1007035. 29718996 PMC5951591

[27] Alonso AM, Corvi MM, Diambra L 2019 Gene target discovery with network analysis in *Toxoplasma gondii*. Sci Rep 9:646. doi:10.1038/s41598-018-36671-y. 30679502 PMC6345969

[28] Watts E, Zhao Y, Dhara A, Eller B, Patwardhan A, Sinai AP 2015 Novel approaches reveal that *Toxoplasma gondii* bradyzoites within tissue cysts are dynamic and replicating entities *in vivo*. mBio 6:e01155-15. doi:10.1128/mBio.01155-15. 26350965 PMC4600105

[29] Murata K, Wolf M 2018 Cryo-electron microscopy for structural analysis of dynamic biological macromolecules. Biochim Biophys Acta Gen Subj 1862:324–334. doi:10.1016/j.bbagen.2017.07.020. 28756276

[30] Sugi T, Kato K, Kobayashi K, Watanabe S, Kurokawa H, Gong H, Pandey K, Takemae H, Akashi H 2010 Use of the kinase inhibitor analog 1NM-PP1 reveals a role for *Toxoplasma gondii* CDPK1 in the invasion step. Eukaryot Cell 9:667–670. doi:10.1128/EC.00351-09. 20173034 PMC2863409

[31] Weiss LM, Laplace D, Takvorian PM, Tanowitz HB, Cali A, Wittner M 1995 A cell culture system for study of the development of *Toxoplasma gondii* bradyzoites. J Eukaryot Microbiol 42:150–157. doi:10.1111/j.1550-7408.1995.tb01556.x. 7757057

[32] Sugi T, Ma YF, Tomita T, Murakoshi F, Eaton MS, Yakubu R, Han B, Tu V, Kato K, Kawazu S-I, Gupta N, Suvorova ES, White MW, Kim K, Weiss LM 2016 *Toxoplasma gondii* cyclic AMP-dependent protein kinase subunit 3 is involved in the switch from tachyzoite to bradyzoite development. mBio 7:e00755-16. doi:10.1128/mBio.00755-16. 27247232 PMC4895117

