# Supplementary figures and images for: The Toxoplasma gondii Cyst Wall Interactome
Source: mBio. 2020 Feb 4;11(1):e02699-19. doi: 10.1128/mBio.02699-19 (PMC7002340; doi:10.1128/mBio.02699-19)

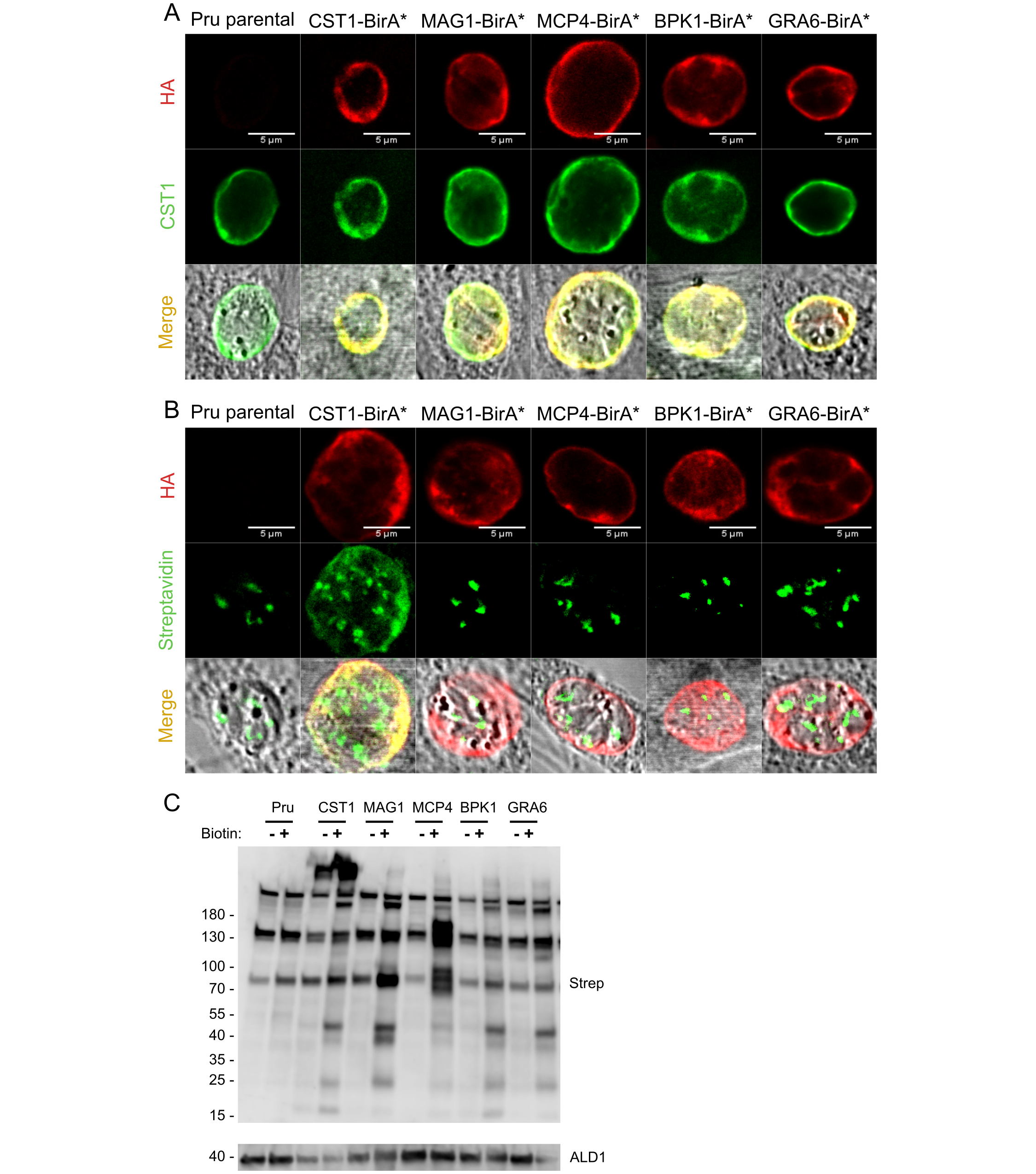

Supplement: FIG S1 [file mBio.02699-19-sf001.tif]

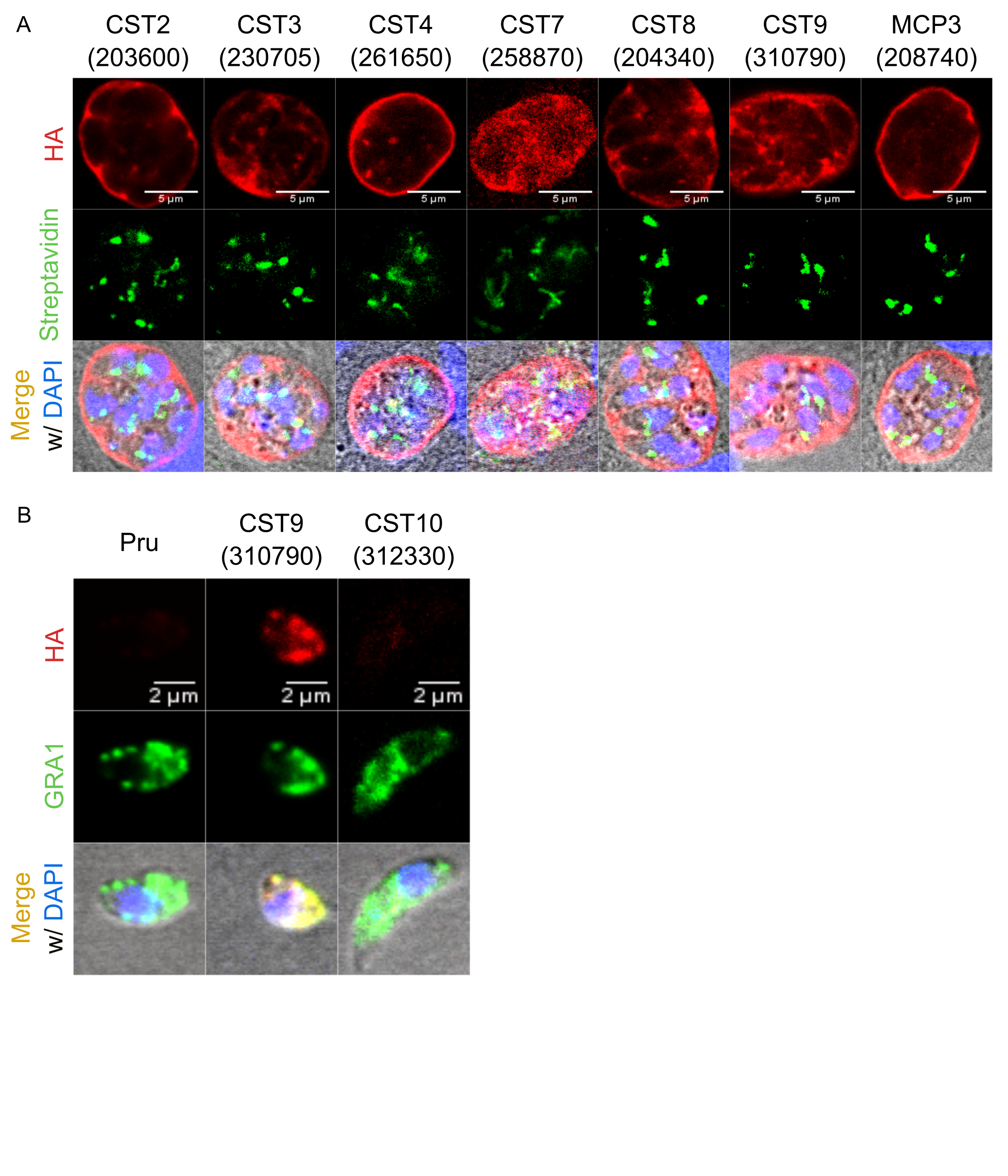

Supplement: FIG S2 [file mBio.02699-19-sf002.tif]

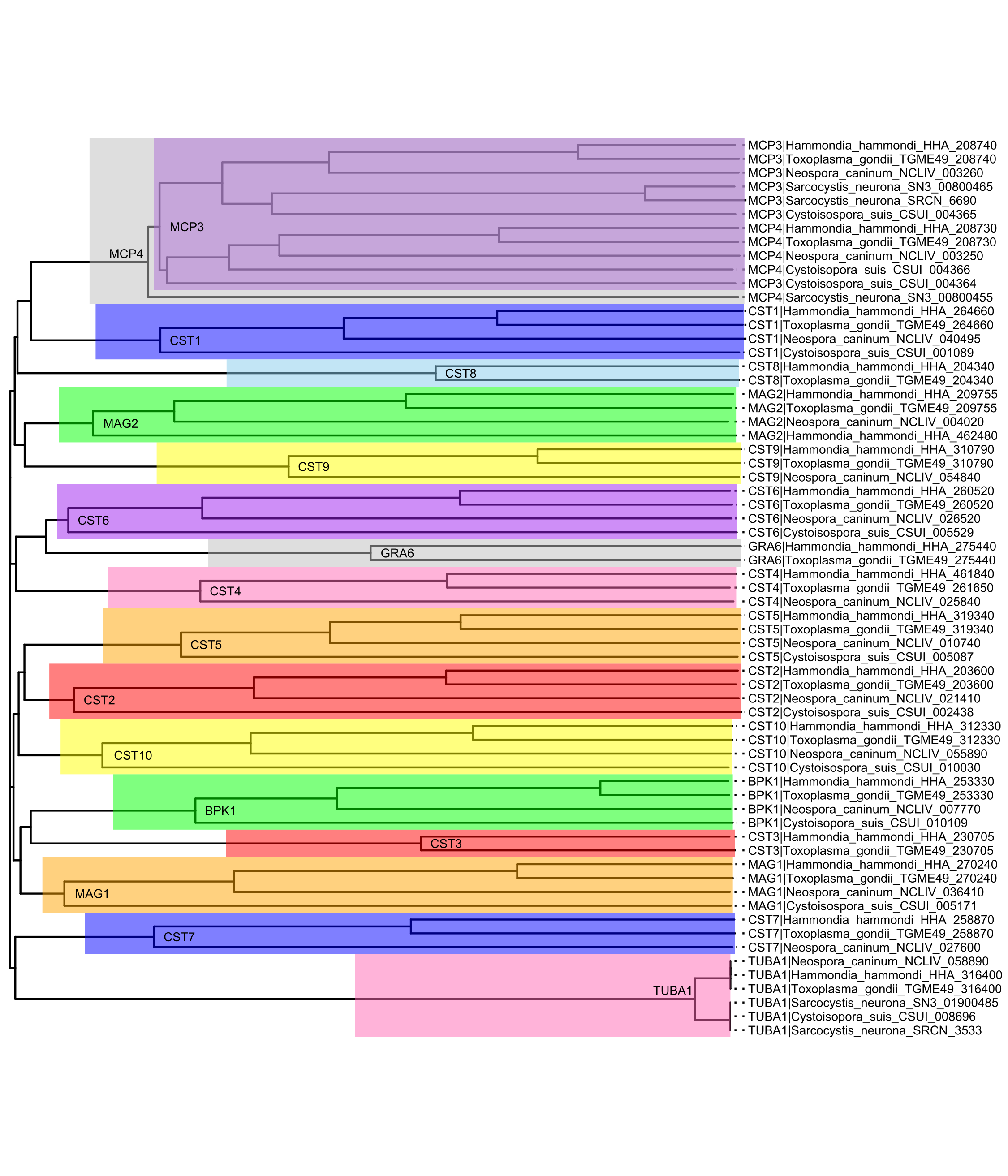

Supplement: FIG S3 [file mBio.02699-19-sf003.tif]

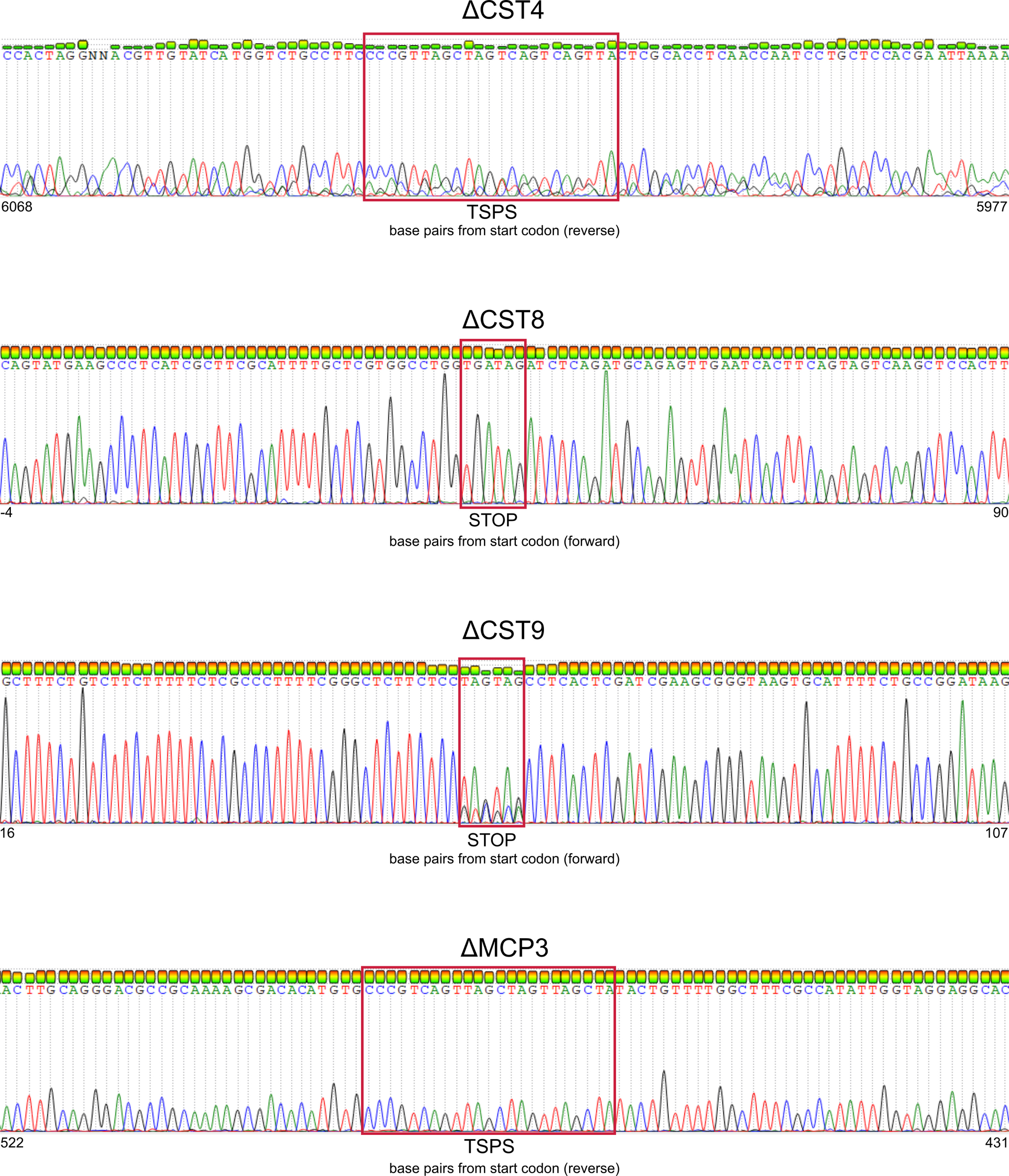

Supplement: FIG S4 [file mBio.02699-19-sf004.jpg]

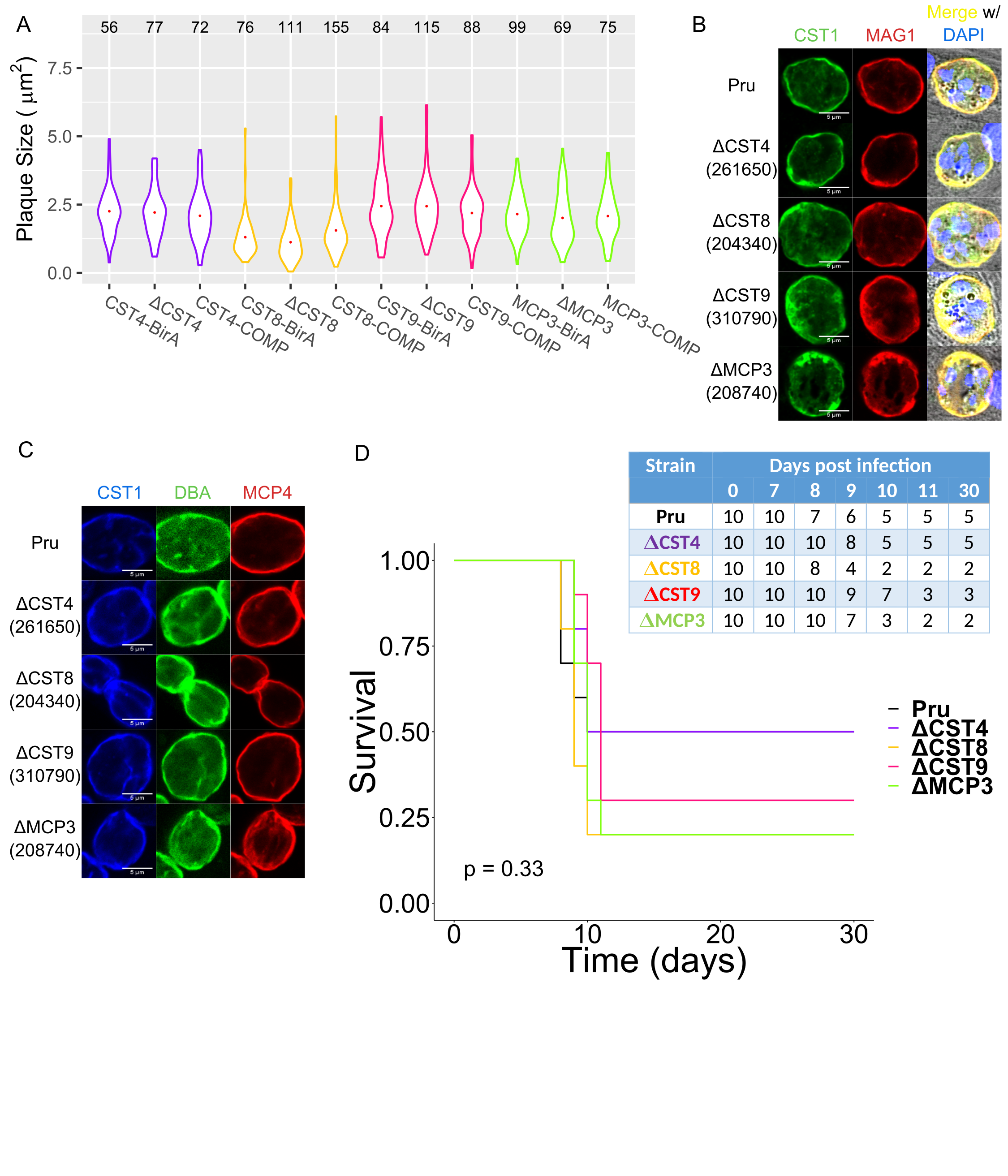

Supplement: FIG S5 [file mBio.02699-19-sf005.tif]
