# Supplementary material for: The Toxoplasma gondii Cyst Wall Interactome
Source: mBio. 2020 Feb 4;11(1):e02699-19. doi: 10.1128/mBio.02699-19 (PMC7002340; doi:10.1128/mBio.02699-19)
Supplement: TABLE S2 [file mBio.02699-19-st002.docx]

| Name | Sequence |
| --- | --- |
| 312875_HA_FWD | CTTTCCTTCAGGTCCCCCGCTTTCGGCGATGCTGCATGCAGTGTACCCTTACGATGTACCGGATTACGCATGAGAACATTCAAAACGCATCTGACCATCT |
| 312875_HA_RVS | AGATGGTCAGATGCGTTTTGAATGTTCTCATGCGTAATCCGGTACATCGTAAGGGTACACTGCATGCAGCATCGCCGAAAGCGGGGGACCTGAAGGAAAG |
| 313080_HA_FWD | CGCCTTCGCCGGAGACTACAAGAAACACAAGGCGTACCCTTACGATGTACCGGATTACGCATAGAAGGGCAGAGAGATTCCGGACTGGGGCAAACAACGG |
| 313080_HA_RVS | CCGTTGTTTGCCCCAGTCCGGAATCTCTCTGCCCTTCTATGCGTAATCCGGTACATCGTAAGGGTACGCCTTGTGTTTCTTGTAGTCTCCGGCGAAGGCG |
| CST10_HA_FWD | TCTCAGGCGTTCGAAAAAGAACAAGAAGAAGACCAGATACCCTTACGATGTACCGGATTACGCATGACAGGAATGTGTCACGTAACTGAAGGGACGTTTT |
| CST10_HA_RVS | AAAACGTCCCTTCAGTTACGTGACACATTCCTGTCATGCGTAATCCGGTACATCGTAAGGGTATCTGGTCTTCTTCTTGTTCTTTTTCGAACGCCTGAGA |
| CST4_KO_TSPS_FWD2 | GTTTTTTAATTCGTGGAGCAGGATTGGTTGAGGTGCGAGtaaCtgaCtgaCtagCtaaCgggGAAGGCAGACCATGATACAACGTTGCCTAGTGGCTTGC |
| CST4_KO_TSPS_RVS2 | GCAAGCCACTAGGCAACGTTGTATCATGGTCTGCCTTCcccGttaGctaGtcaGtcaGttaCTCGCACCTCAACCAATCCTGCTCCACGAATTAAAAAAC |
| CST8_KO_FWD | CTTCGCATTTTGCTCGTGGCCTGGtgatagATCTCAGATGCAGAGTTGAATCACTTCAGTAGTCAAGCTCCACTTAGCTGGCCAAGGTGTGTCGTCGTGT |
| CST8_KO_RVS | ACACGACGACACACCTTGGCCAGCTAAGTGGAGCTTGACTACTGAAGTGATTCAACTCTGCATCTGAGATctatcaCCAGGCCACGAGCAAAATGCGAAG |
| CST9_KO_FWD | TGCTTTCTGTCTTCTTTTTCTCGCCCTTTTCGGGCTCTTCTCCtagtagCCTCACTCGATCGAAGCGGGTAAGTGCATTTTCTGCCGGATAAGTCGCCGA |
| CST9_KO_RVS | TCGGCGACTTATCCGGCAGAAAATGCACTTACCCGCTTCGATCGAGTGAGGctactaGGAGAAGAGCCCGAAAAGGGCGAGAAAAAGAAGACAGAAAGCA |
| MCP3_KO_TSPS_FWD2 | TGGTGCCTCCTACCAATATGGCGAAAGCCAAAACAGTAtagCtaaCtagCtaaCtgaCgggCACATGTGTCGCTTTTGCGGCGTCCCTGCAAGTTGCTGT |
| MCP3_KO_TSPS_RVS2 | ACAGCAACTTGCAGGGACGCCGCAAAAGCGACACATGTGcccGtcaGttaGctaGttaGctaTACTGTTTTGGCTTTCGCCATATTGGTAGGAGGCACCA |
| CST4_COMP _FWD | TTGTGTTGTTTTTTAATTCGTGGAGCAGGATTGGcttAGGTGCGAGACCACTGTTGTGAGCGGAgagCAGAAGGCAGACCATGATACAACGTTGCCTAGT |
| CST4_COMP_RVS | ACTAGGCAACGTTGTATCATGGTCTGCCTTCTGctcTCCGCTCACAACAGTGGTCTCGCACCTaagCCAATCCTGCTCCACGAATTAAAAAACAACACAA |
| CST8_COMP_FWD | CTTCGCATTTTGCTCGTGgcgTGGACGATCATCTCAGATGCAGAGTTGAATCACTTCAGTAGTCAAGCTcctctgtcgTGGCCAAGGTGTGTCGTCGTGT |
| CST8_COMP_RVS | ACACGACGACACACCTTGGCCAcgacagaggAGCTTGACTACTGAAGTGATTCAACTCTGCATCTGAGATGATCGTCCAcgcCACGAGCAAAATGCGAAG |
| CST9_COMP_FWD | TGCTTTCTGTCTTCTTTTTCTCGCCCTTTTCGGGCTCTTCagtGTCGCACCTcatagcATCGAAGCGGGTAAGTGCATTTTCTGCCGGATAAGTCGCCGA |
| CST9_COMP_RVS | TCGGCGACTTATCCGGCAGAAAATGCACTTACCCGCTTCGATgctatgAGGTGCGACactGAAGAGCCCGAAAAGGGCGAGAAAAAGAAGACAGAAAGCA |
| MCP3_COMP_FWD | TCCCCCTGGTGCCTCCTACCAATATGGCGAAAGCCaagACAGTAGGAGTGCGCCTGATTGGCgtcACCACATGTGTCGCTTTTGCGGCGTCCCTGCAAGT |
| MCP3_COMP_RVS | ACTTGCAGGGACGCCGCAAAAGCGACACATGTGGTgacGCCAATCAGGCGCACTCCTACTGTcttGGCTTTCGCCATATTGGTAGGAGGCACCAGGGGGA |

**Supplemental Table S2**

Oligonucleotides used as donor DNA to generate HA tagged, knockout, and complement strains. Mutations are designated in red with stop codons designated as lowercase letters and HA sequence in green.
