# Supplementary material for: The Toxoplasma gondii Cyst Wall Interactome
Source: mBio. 2020 Feb 4;11(1):e02699-19. doi: 10.1128/mBio.02699-19 (PMC7002340; doi:10.1128/mBio.02699-19)
Supplement: TABLE S1 [file mBio.02699-19-st001.docx]

| Name | Sequence | Purpose |
| --- | --- | --- |
| BirA_FWD | GACAAGGACAACACCGTGCC | Inserting BirA* into pLIC |
| BirA_GA_RVS | GGGACGTCGTACGGGTACCTCTTCTCTGCGCTTCTCAGGG | Inserting BirA* into pLIC |
| pLIC_3HA_FWD | AGGTACCCGTACGACGTC | pLIC amplification |
| pLIC_3HA_RVS | TTCCGAGCTTGGCGTAATCA | pLIC amplification |
| BPK1_GA_FWD | TGATTACGCCAAGCTCGGAATTGAGAGGAACGTTTTCCGA | Inserting BPK1 into BirA*-pLIC |
| BPK1_GA_RVS | GGCACGGTGTTGTCCTTGTCCAGACGGTTCTGCTTGTACC | Inserting BPK1 into BirA*-pLIC |
| MCP4_GA_FWD | TGATTACGCCAAGCTCGGAAATGTGAACCCCCTCACACTA | Inserting MCP4 into BirA*-pLIC |
| MCP4_GA_RVS | GGCACGGTGTTGTCCTTGTCTTTACTGGTAGGATTTCGCC | Inserting MCP4 into BirA*-pLIC |
| MAG1_GA_FWD | TGATTACGCCAAGCTCGGAAGACGTGTCGAAACAAGCTGA | Inserting MAG1 into BirA*-pLIC |
| MAG1_GA_RVS | GGCACGGTGTTGTCCTTGTCAGCTGCCTGTTCCGCTAAGA | Inserting MAG1 into BirA*-pLIC |
| GRA6_GA_FWD | GATTACGCCAAGCTCGGAAAACCACTCGAATTTTACGATA | Inserting GRA6 into BirA*-pLIC |
| GRA6_GA_RVS | GGCACGGTGTTGTCCTTGTCAAAATCAAACTCATTCACAC | Inserting GRA6 into BirA*-pLIC |
| DHFR_check_RVS | ACGGCGTTGAATCTCTTG | DHFR sequence check |
| Gene_5UTR_check_FWD | AGCGGATAACAATTTCACACAGGA | Gene 5’UTR check |
| BirA_check_RVS | CTCAGTGTCTGGATGTGCTTG | Gene C-terminus check |
| BirA_3HA_HXGPRT_RVS | TTATCGATACCGTCGAGGGGGGTAGTGGATCCGAGCACGC | BirA*-3xHA-HXGPRT 3UTR amplification |
| DHFR_FWD | CCCCCCTCGACGGTATCGAT | DHFR amplification |
| DHFR_RVS | GCAAACGCTTCCGCAAAGGT | DHFR amplification |
| pUC19_FWD | ATTTAAATGGATCCTCTAGAGTCGACCTGC | pUC19 amplification |
| pUC19_RVS | ATTTAAATTACCGAGCTCGAATTCACTGG | pUC19 amplification |
| CST1_Cterm_GA_FWD | ATTCGCCATTTAAATGCCTGTGGTTTTCGAGCTTCCTTCC | CST1-Cterm amplification |
| CST1_Cterm_GA_RVS | GTCCTTGTCCTCATGTCCGCAACAACAACAGCCT | CST1-Cterm amplification |
| CST1_3UTR_GA_FWD | CGTAATAGCGAAGAGGCCCGAGGCGCCGCCGCTACAGTTT | CST1-3UTR amplification |
| CST1_3UTR_GA_RVS | TCTAGAGGATCCATTTAAATTGCTGGTTGGATGCCGCTGA | CST1-3UTR amplification |
| CST2_Cterm_GA_FWD | TCGAGCTCGGTAATTTAAATCCGCCACCTATTGGTGTGAT | Inserting CST2-C-term into BirA* pUC19 |
| CST2_Cterm_GA_RVS | GGCACGGTGTTGTCCTTGTCTGCCTTATTATCGCAGCAGC | Inserting CST2-C-term into BirA* pUC19 |
| CST2_3UTR_GA_FWD | ACCTTTGCGGAAGCGTTTGCGTAGATGGCTAATGCCGCGA | Inserting CST2-3UTR into BirA* pUC19 |
| CST2_3UTR_GA_RVS | TCTAGAGGATCCATTTAAATACACAGCTCCCTTGAGTTCG | Inserting CST2-3UTR into BirA* pUC19 |
| CST3_Cterm_GA_FWD | TCGAGCTCGGTAATTTAAATTGCACTTAAAAGGCCAGGTG | Inserting CST3-C-term into BirA* pUC19 |
| CST3_Cterm_GA_RVS | GGCACGGTGTTGTCCTTGTCTTTCGGTTCACGCGACTCCG | Inserting CST3-C-term into BirA* pUC19 |
| CST3_3UTR_GA_FWD | ACCTTTGCGGAAGCGTTTGCACGAGCGGTCGAAGTGTAAA | Inserting CST3-3UTR into BirA* pUC19 |
| CST3_3UTR_GA_RVS | TCTAGAGGATCCATTTAAATGGAACGAAGTCGACTCGGAA | Inserting CST3-3UTR into BirA* pUC19 |
| CST4_Cterm_GA_FWD | TCGAGCTCGGTAATTTAAATATCGTACGTGACTCAGTGCC | Inserting CST4-C-term into BirA* pUC19 |
| CST4_Cterm_GA_RVS | CACGGTGTTGTCCTTGTCGTCCTCCTCAGCGATAAAAAAG | Inserting CST4-C-term into BirA* pUC19 |
| CST4_3UTR_GA_FWD | ACCTTTGCGGAAGCGTTTGCAACGCAGATTCCGTCCCAAT | Inserting CST4-3UTR into BirA* pUC19 |
| CST4_3UTR_GA_RVS | TCTAGAGGATCCATTTAAATCTACGCGCCGATTTCCTAGT | Inserting CST4-3UTR into BirA* pUC19 |
| CST7_Cterm_GA_FWD | TCGAGCTCGGTAATTTAAATGAGCGACGTATGAGTAAGCA | Inserting CST7-C-term into BirA* pUC19 |
| CST7_Cterm_GA_RVS | GGCACGGTGTTGTCCTTGTCCTGCACAACCAAGAAAACAA | Inserting CST7-C-term into BirA* pUC19 |
| CST7_3UTR_GA_FWD | ACCTTTGCGGAAGCGTTTGCTTGAGGGAGTGACGTTTCCG | Inserting CST7-3UTR into BirA* pUC19 |
| CST7_3UTR_GA_RVS | TCTAGAGGATCCATTTAAATTGACTTGATCGCCTGTGCTT | Inserting CST7-3UTR into BirA* pUC19 |
| CST8_Cterm_GA_FWD | TCGAGCTCGGTAATTTAAATCATAGTGTATATCGGAGTTA | Inserting CST8-C-term into BirA* pUC19 |
| CST8_Cterm_GA_RVS | GGCACGGTGTTGTCCTTGTCATACAAAAAACTGAGATAAG | Inserting CST8-C-term into BirA* pUC19 |
| CST8_3UTR_GA_FWD | ACCTTTGCGGAAGCGTTTGCCTGAGGACTGCCAACACTGG | Inserting CST8-3UTR into BirA* pUC19 |
| CST8_3UTR_GA_RVS | TCTAGAGGATCCATTTAAATGATACCTGACTGCAGGACGC | Inserting CST8-3UTR into BirA* pUC19 |
| CST9_Cterm_GA_FWD | TCGAGCTCGGTAATTTAAATAGCTACCACGAAGCACTTCT | Inserting CST9-C-term into BirA* pUC19 |
| CST9_Cterm_GA_RVS | GGCACGGTGTTGTCCTTGTCCAGCTCCTCCGCCTTCTCTT | Inserting CST9-C-term into BirA* pUC19 |
| CST9_3UTR_GA_FWD | ACCTTTGCGGAAGCGTTTGCTTTCCTTCTTGTTTGCGGT | Inserting CST9-3UTR into BirA* pUC19 |
| CST9_3UTR_GA_RVS | TCTAGAGGATCCATTTAAATATCTTGATTTCTGCGCGT | Inserting CST9-3UTR into BirA* pUC19 |
| MCP3_Cterm_GA_FWD | TCGAGCTCGGTAATTTAAATCCTACCACAGGTGCAGACAT | Inserting MCP3-C-term into BirA* pUC19 |
| MCP3_Cterm_GA_RVS | GGCACGGTGTTGTCCTTGTCGTATCCCCGTAGCCTTCGAT | Inserting MCP3-C-term into BirA* pUC19 |
| MCP3_3UTR_GA_FWD | ACCTTTGCGGAAGCGTTTGCCACGAACGGCTGACAAGAAA | Inserting MCP3-3UTR into BirA* pUC19 |
| MCP3_3UTR_GA_RVS | TCTAGAGGATCCATTTAAATTACCAGTCAGCGACGGTAGT | Inserting MCP3-3UTR into BirA* pUC19 |
| M13_RVS | GGAAACAGCTATGACCATG | Gene 3’UTR check |
| sg312875-HA | GTCACACTGCATGCAGCATCGGTTTTAGAGCTAGAAATAG | HA tag hypothetical protein |
| sg313080-HA | GCAAGAAACACAAGGCGTAGAGTTTTAGAGCTAGAAATAG | HA tag hypothetical protein |
| sgCST10-HA | GCAAGAAGAAGACCAGATGACGTTTTAGAGCTAGAAATAG | HA tag hypothetical protein |
| sgCST4-KO_1 | GAAGAAATCGAACAGATCAGCGTTTTAGAGCTAGAAATAG | guide RNA targeting N-terminus |
| sgCST4-KO_2 | GTGCGAGACCACTGTTGTGAGGTTTTAGAGCTAGAAATAG | guide RNA targeting downstream N-terminus |
| sgCST8-KO | GTGCATCTGAGATGATCGTCCGTTTTAGAGCTAGAAATAG | guide RNA targeting N-terminus |
| sgCST9-KO | GTTCGATCGAGTGAGGTGCGAGTTTTAGAGCTAGAAATAG | guide RNA targeting N-terminus |
| sgMCP3-KO_1 | GTATAGCACCATCTTTCGCGAGTTTTAGAGCTAGAAATAG | guide RNA targeting N-terminus |
| sgMCP3-KO_2 | GACAGTAGGAGTGCGCCTGATGTTTTAGAGCTAGAAATAG | guide RNA targeting downstream N-terminus |
| sgCST4-COMP | GTAACTGACTGACTAGCTAACGTTTTAGAGCTAGAAATAG | guide RNA targeting TSPS |
| sgCST8-COMP | GTAGTCAAGCTCCACTTAGCGTTTTAGAGCTAGAAATAGC | guide RNA targeting KO N-terminus |
| sgCST9-COMP | GTTACCCGCTTCGATCGAGTGGTTTTAGAGCTAGAAATAG | guide RNA targeting KO N-terminus |
| sgMCP3-COMP | GTAGCTAACTAGCTAACTGACGTTTTAGAGCTAGAAATAG | guide RNA targeting TSPS |
| sgRNA_check_FWD | GCTTGCGCAGCATACACTC | sgRNA check on plasmid |
| HXGPRT_check_RVS | AAGCTGCACGCACATGAAAT | HXGPRT check on plasmid |
| Cas9_check_RVS | CACCAGTTTCTTTCTCAGGTGGT | Cas9 check on plasmid |

**Supplemental Table S1**

All primers used in this study. Overhanging regions for Gibson Assembly or KLD reaction are designated in green.
